# Do biosurfactants as anti-biofilm agents have a future in industrial water systems?

**DOI:** 10.3389/fbioe.2023.1244595

**Published:** 2023-09-15

**Authors:** Abdullahi Adekilekun Jimoh, Elzaan Booysen, Leonardo van Zyl, Marla Trindade

**Affiliations:** Department of Biotechnology, Institute for Microbial Biotechnology and Metagenomics (IMBM), University of the Western Cape, Cape Town, South Africa

**Keywords:** biocorrosion, biocides, biofilms, biofouling, dispersants, industrial wastewater management

## Abstract

Biofilms are bacterial communities embedded in exopolymeric substances that form on the surfaces of both man-made and natural structures. Biofilm formation in industrial water systems such as cooling towers results in biofouling and biocorrosion and poses a major health concern as well as an economic burden. Traditionally, biofilms in industrial water systems are treated with alternating doses of oxidizing and non-oxidizing biocides, but as resistance increases, higher biocide concentrations are needed. Using chemically synthesized surfactants in combination with biocides is also not a new idea; however, these surfactants are often not biodegradable and lead to accumulation in natural water reservoirs. Biosurfactants have become an essential bioeconomy product for diverse applications; however, reports of their use in combating biofilm-related problems in water management systems is limited to only a few studies. Biosurfactants are powerful anti-biofilm agents and can act as biocides as well as biodispersants. In laboratory settings, the efficacy of biosurfactants as anti-biofilm agents can range between 26% and 99.8%. For example, long-chain rhamnolipids isolated from *Burkholderia thailandensis* inhibit biofilm formation between 50% and 90%, while a lipopeptide biosurfactant from *Bacillus amyloliquefaciens* was able to inhibit biofilms up to 96% and 99%. Additionally, biosurfactants can disperse preformed biofilms up to 95.9%. The efficacy of antibiotics can also be increased by between 25% and 50% when combined with biosurfactants, as seen for the V9T14 biosurfactant co-formulated with ampicillin, cefazolin, and tobramycin. In this review, we discuss how biofilms are formed and if biosurfactants, as anti-biofilm agents, have a future in industrial water systems. We then summarize the reported mode of action for biosurfactant molecules and their functionality as biofilm dispersal agents. Finally, we highlight the application of biosurfactants in industrial water systems as anti-fouling and anti-corrosion agents.

## 1 Introduction

**FIGURE 1:**
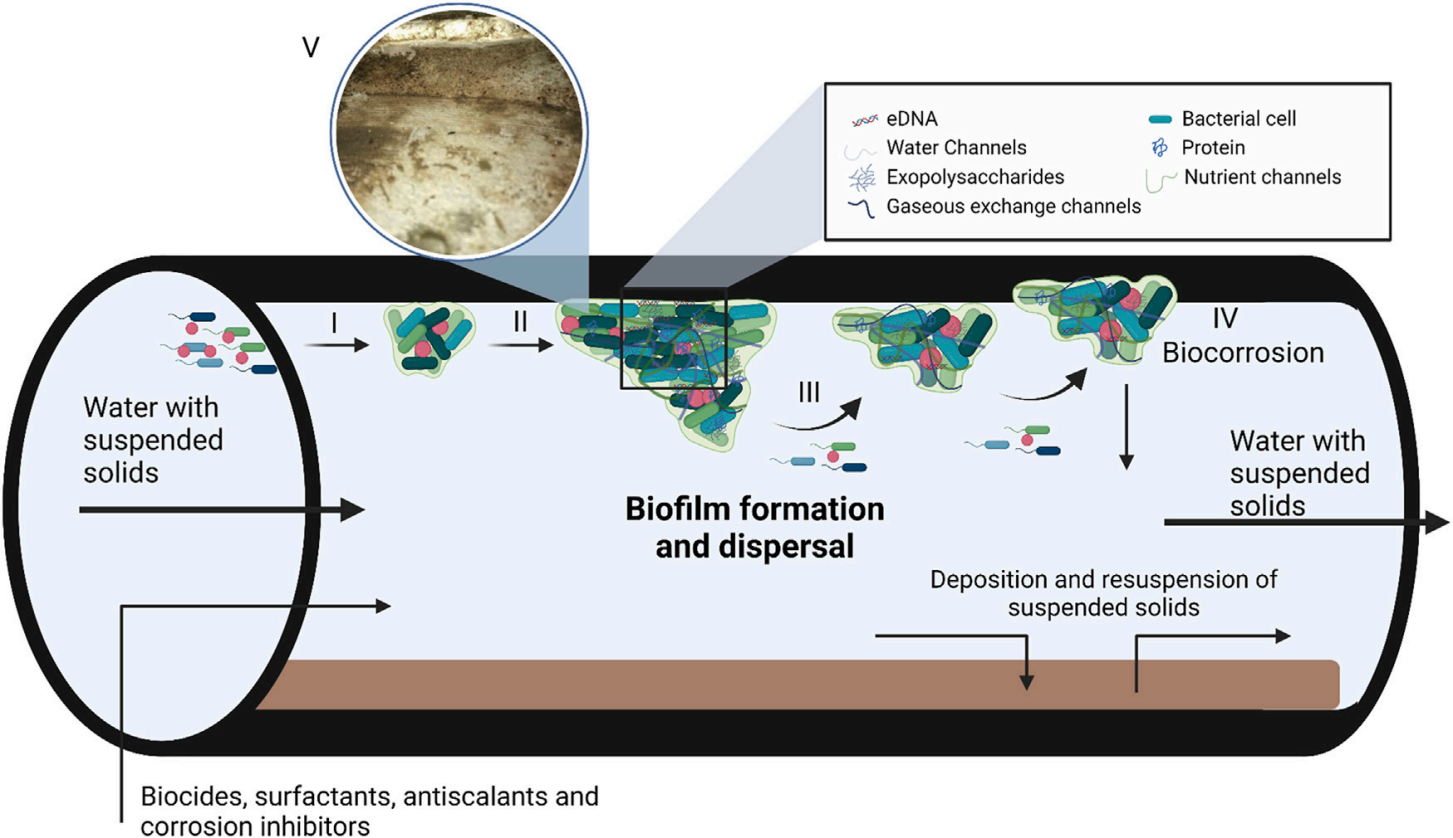
A model depicting biofilm formation and composition in steel pipes used in industrial water systems. Biofilm formation takes place in three steps, i.e., adsorption (I), maturation (II) and dispersion (III). During the adsorption step, planktonic bacteria use cellular appendages to overcome the repulsive forces of surfaces, followed by the production of the extracellular matrix (ECM). As the bacteria produce the ECM and the population density increases gene expression is altered by quorum sensing. Once the biofilm matures and reaches its critical mass, the biofilm enters the dispersion phase to form new biofilms. The ECM consist of DNA, polysaccharides and proteins with various water, nutrient and gaseous exchange channels within the matrix. (IV) Biofilm maturation can also lead to corrosion of the industrial systems, known as biocorrosion. (V) Insert of a biofilm that formed in a cooling tower at a South African Brewery, the image was kindly supplied by Biodx (pty) ltd. Figure adapted from Verderosa et al. (2019); Rabin et al. (2015) and Verberk et al. (2009). Created with BioRender.com.

Biofilms are sessile microbial communities embedded in a self-produced extracellular matrix (ECM) attached to abiotic and biotic surfaces (Amirinejad et al., 2023; Ng et al., 2023). The ECM consists of varying ratios of polysaccharides, proteins and nucleic acids depending on microbial composition and environment of the biofilm (Figure 1) (Okuda et al., 2018). Biofilms are of great concern and have detrimental impacts on healthcare, industrial manufacturing, food processing and packaging, thermoelectric, mining, and marine industries (Muhammad et al., 2020; Shineh et al., 2023). As an example, biofilms can cause persistent infections when formed on medical devices or cause nosocomial infections when formed on hospital surfaces such as sink drains and pipes (Dongari-Bagtzoglou, 2008; Percival et al., 2015). The impact that biofilms have on the food industry has negative health and economic effects, as seen with the recent *Listeria monocytogenes* outbreak in South Africa (Colagiorgi et al., 2017; Zhang et al., 2021). Also, the biofilm communities multiply and colonize the surfaces of most industrial water systems (Pereira et al., 2017; Di Pippo et al., 2018; Wang et al., 2023). Water cooling towers are the industrial water systems most affected by biofilm formation, biofouling, and biocorrosion. Cooling towers are used in many industries to dissipate heat, including steel mills, refineries, petrochemicals, food—and power plants (Liu et al., 2011; Di Pippo et al., 2018).

The high nutrient load, warmer than ambient temperature, neutral pH and continuous aeration make water cooling towers the ideal environment for many pathogenic bacteria (Wéry et al., 2008); see Di Pippo et al. (2018) for a list of main microbial species found in water cooling towers. Although many pathogenic microbial species are associated with water cooling towers, *Legionella pneumophila* is of huge concern. This strain is the major causative agent of legionnaires’ disease, a severe multisystem disease involving pneumonia with a case fatality rate of 10%–15% (Walser et al., 2014). In less severe cases, infection of *Legionella* spp. manifests as Pontiac fever presenting with flu-like symptoms (Diederen, 2008). *Legionella* spp. are facultative intracellular Gram-negative bacilli that live within microbial biofilm communities, making them notoriously difficult to eradicate (Fields et al., 2002). Therefore, eliminating microbial biofilm formation can drastically reduce the population of *Legionella* spp. in cooling water towers.

Biofilms in cooling water towers also have a negative financial impact. They cause accelerated metal corrosion, increased resistance to heat transfer, and increased fluid frictional resistance, effectively decreasing the efficacy of the cooling tower (Cloete et al., 1998). Traditionally, a combination of continuous oxidizing biocides and periodic doses of non-oxidizing biocide is used to treat biofilm formation in industrial water systems (Liu et al., 2011). Biocides are substances that can kill, destroy, inhibit, or control the growth of microbial organisms (Makhlouf and Botello, 2018). Chemical surfactants have become essential constituents to enhance biocide effectivity (Simões et al., 2005). In particular, the combination of chemical surfactants with biocides offers a significant improvement as these surface-active compounds form micelles. These micelles act as wetting agents by increasing the penetrating properties of the surfactants, making them more effective at disrupting the biofilm matrix (Percival et al., 2019). A few drawbacks are associated with this treatment approach, including environmental contamination when runoff water enters natural water reservoirs, increasing concentrations needed to ensure effectivity and increased costs related to the need for higher concentrations (Di Pippo et al., 2018).

Increasing environmental legislation puts pressure on the water treatment industry to find alternative sources or significantly reduce the concentration of chemical biocides (Cloete and Flemming, 2012). One proposed solution is using biosurfactants as alternative biocides or in co-formulation with chemical biocides. Biosurfactants are amphipathic bioactive compounds from natural sources and are an alternative to petroleum-based surfactants (Costa et al., 2018). Biosurfactants can be structurally classified into many groups, and their effectiveness is determined by analyzing their hydrophilic-lipophilic balance (Pacwa-Plociniczak et al., 2011). In contrast to petroleum-based surfactants, biosurfactants have several advantages, including lower toxicity, higher specificity, improved biodegradability, superior foaming properties and improved effectiveness and stability at extreme pH, temperatures, and salinity (Kosaric, 2001). In addition to reducing surface tension, some biosurfactants have inherent antimicrobial and dispersal activity (De Giani et al., 2021). Currently, biosurfactants play a minor role in water management, and this review aims to assess their capacity to function as anti-biofilm agents for industrial water systems and other water management applications concerning biofilm control and disruption, microbial fouling, and associated corrosion.

## 2 Biofilm formation

Biofilms form in response to changes in environmental conditions, such as limited nutrients, desiccation, extreme pH, ultraviolet radiation, extreme temperatures, antimicrobial agents, high atmospheric pressure, and high salt concentrations (O’Toole et al., 2000). Biofilm formation is a multi-step process characterized by initial adsorption, maturation, and dispersion (Figure 1). The adsorption step is further divided into reversible and irreversible attachment (Renner and Weibel, 2011). During the initial reversible attachment stage, cellular appendages such as flagella, fimbriae, glycocalyx, and pili play an essential role in overcoming the repulsive forces common to most surfaces (Rosenberg et al., 1982; Korber et al., 1989; Donlan, 2001; Palmer et al., 2007). The production of exopolysaccharides (EPS) signifies the irreversible attachment of the biofilm to the surface and allows the biofilm to mature (Rabin et al., 2015). Once the biofilm reaches a critical mass, it will start to actively disperse to form a new biofilm elsewhere. Dispersion caused by external sources such as biocides, antibiotics and biosurfactants is termed passive dispersal (Kaplan, 2010). The production of EPS, higher cell density of heterogeneous bacterial communities and attachment to the surface also result in changes in gene expression and growth rate of sessile bacteria (Flemming et al., 2007). The accumulation of signalling molecules involved in quorum sensing in the extracellular environment also plays a significant role in gene expression, biofilm formation, and dispersion (Solano et al., 2014).

Exopolysaccharides form the structural base and are attached to the cell surface creating large networks that serve as scaffolds for proteins, nucleic acids, lipids, and carbohydrates (Rabin et al., 2015). The composition, properties and structure of the exopolysaccharides can differ substantially between individual bacterial species as well as microbial consortia. The monomers galactose, mannose and glucose are the most abundant carbohydrates, followed by galacturonic acid, arabinose, fucose, xylose, rhamnose and *N-*acetyl-glucosamine (Bales et al., 2013). Many of the polysaccharides that compose biofilms are produced at all microbial growth stages; however, a significant increase in their production is observed in bacteria entrapped in a biofilm such as colanic acid, alginate, Pel and Psl. Colanic acid is found in biofilms of *Enterobacteriaceae* (Prigent-Combaret et al., 1999), and it consists of repeated units of L-fucose, D-galactose, D-glucose and D-glucuronate with *O-*acetyl and pyruvate side chains (Stevenson et al., 1996). Colanic acid is assembled and excreted by the Wzx system; WzC and WzB play a role in polymerization, while WzA transports colanic acid across the membrane (Reid and Whitfield, 2005). Although colanic acid plays a significant role in biofilm formation by *Enterobacteriaceae* it is not essential, as seen in *Escherichia coli* K-12 strains that are defective in colanic acid production yet still able to form biofilms, albeit at a slower rate (Danese et al., 2000). Alginate, Pel and Psl are polysaccharides associated with *Pseudomonas aeruginosa* biofilm formation. Psl is a mannose-rich exopolysaccharide produced by mucoid-positive *P. aeruginosa* strains such as ZK2870 and PA01, while Pel is a glucose-rich exopolysaccharide produced by the mucoid-negative strain *P. aeruginosa* PA14, and alginate is produced by mucoid rich strains that colonize the lungs of cystic fibrosis patients (Colvin et al., 2012).

In addition to exopolysaccharides, extracellular proteins (Eps) help to stabilize and form biofilms. One example is glucan-binding proteins (Gbps) found in *Streptococcus mutans* biofilms. Lynch and co-authors found that Gbps play a crucial role in maintaining the architecture of *S. mutans* biofilms by linking bacteria and exopolysaccharides (Lynch et al., 2007). Another example of Eps is amyloids such as Fap amyloids found in the EPS of *Pseudomonas* spp. and TasA amyloids found in *Bacillus subtilis*. Dueholm and co-authors found that when Fap amyloids were overexpressed in *Pseudomonas* spp., cell aggregation and biofilm formation increased (Dueholm et al., 2013), while Romero and co-authors found that TasA amyloids play an important role in the structural integrity of *B. subtilis* biofilms (Romero et al., 2010). Not all Eps play a role in biofilm formation and structural integrity, as some enzymes are involved in biofilm degradation, detachment, and dispersal. These enzymes are crucial in releasing nutrients during starvation (Zhang and Bishop, 2003) and initiating a new biofilm lifecycle such as DspB in *Actinobacillus pleuropneumoniae* (Kaplan et al., 2004).

Initially, researchers assumed that extracellular DNA (eDNA) is leftovers from lysed cells, but Whitchurch and co-authors demonstrated that eDNA is essential to biofilm formation (Whitchurch et al., 2002). While the negative charges of DNA act as a repulsive force during the initial attachment, the eDNA starts to facilitate adhesion by interacting with the receptors on nearby bacteria once the distance decreases to a few nanometers (Das et al., 2010). Gloag and co-authors also showed that eDNA promotes *P. aeruginosa* biofilm expansion by coordinating cell movement (Gloag et al., 2013). Numerous studies have also shown that eDNA can increase antibiotic resistance in biofilms by either inhibiting transport of antibiotics within the biofilm (Doroshenko et al., 2014) or by activating the PhoPQ/PmrAB two-component system (Mulcahy et al., 2008; Johnson et al., 2013; Lewenza, 2013). Numerous other examples of extracellular saccharides, proteins and DNA also exist, as shown in a comprehensive review by (Rabin et al., 2015).

### 2.1 Tolerance to antibiotics

Biofilms are notoriously difficult to treat and are up to 1000 times more resistant to antibiotics than their planktonic counterparts (Sharma et al., 2019). Multiple factors contribute to antibiotic resistance exhibited by bacterial biofilms. These factors include limited antibiotic and biocide penetration, efflux pumps, persister cells, reduced growth rate and horizontal gene transfer (Verderosa et al., 2019). A biofilm’s first line of defense against antibiotics is the EPS matrix, which is achieved by limiting the penetration of antibiotics into the biofilm. As mentioned above, eDNA can chelate numerous antibiotics, thus trapping the antibiotics and preventing them from moving through the EPS matrix. Numerous studies have shown that a steep gradient difference in antibiotic concentration is observed when the concentration of antibiotics at the base of the biofilm is compared to the outer regions of the biofilm (Anderl et al., 2000; Doroshenko et al., 2014; Justo and Bookstaver, 2014). Another advantage is high cell density that facilitates horizontal gene transfer and effectively increases the spread of plasmid-borne antibiotic resistance genes, as demonstrated in *S. aureus* (Savage et al., 2013). Most antibiotics target actively growing and dividing cells; thus, the slow metabolic rate of bacteria deep within the biofilm protects them against antibiotics that do manage to penetrate the EPS (Ashby et al., 1994). Persister cells employ the same method to ensure survival and act as disease reservoirs once the antibiotic pressure is removed (Keren et al., 2004; Lewis, 2007). Although efflux pumps are present in planktonic cells, Zhang and co-authors found that efflux pumps involved in antibiotic resistance are upregulated in biofilms (Zhang and Mah, 2008). The Verderosa et al. (2019) review provides a more comprehensive summary of how biofilms evade antibiotics.

### 2.2 Biofilms in industrial water systems

Industrial water usage requires water management systems which are usually prone to biofilm development (Figure 1). Petroleum refineries, steel mills, power generation plants, and petrochemical plants often have onsite industrial water management facilities, and biofilm communities colonize the surfaces of most of these as well as associated equipment such as fill material, reservoirs, submerged sight glasses and sensors, heat exchangers and pipelines (Liu et al., 2011; Rao, 2012; Di Pippo et al., 2018). Furthermore, the worldwide shortage of fresh water means that industrial water is constantly recycled, increasing the nutrient load of industrial water systems, thus creating the ideal environment for biofilm formation (Coetser and Cloete, 2005; Flemming et al., 2007). In addition, bacteria can also use some antiscalants and corrosion inhibitors as nutrient sources depending on their composition (Kusnetsov et al., 1993). Mechanical cleaning of cooling towers effectively removes biofilms, but structural design can make this method impossible. Thus, alternative methods are needed to remove biofilms and prevent biofouling in cooling towers (Wang et al., 2023). Various oxidizing (mainly chlorine, calcium hypochlorite, sodium hypochlorite, ozone, hydrogen peroxide, bromine chloride) and/or non-oxidizing (principally heavy metal compounds, amines, aldehydes, thiocyanates, isothiazolone, and organo-bromine compounds) agents are used to prevent microbial growth and microbially induced corrosion in many industrial water system components (Cloete and Flemming, 2012). Microbially-induced corrosion is a significant problem as numerous metals, such as nickel and aluminium-based alloys, comprise the base structure of industrial water-cooling circuits and towers. Exposure of these substances through an electrochemical reaction between the interfaces can lead to corrosion (Figure 1) (Beech et al., 2000).

Biocide treatment regimens are designed to treat wastewater streams for safe discharge into receiving waterbodies (AquaTech, 2019), with chlorine and ozone being the two major biocides used for years. Unfortunately, this process can lead to contamination of drinking water reservoirs and is considered toxic to the environment (Williams and McGinley, 2010). In addition, due to the high tolerance of bacteria in biofilms to these toxic biocides, higher than normal concentrations are needed which increases the cost and the burden on the environment (Maaike et al., 2006). As the biofilm thickens, higher biocide concentrations are required to penetrate the deeper levels and if sub-inhibitory concentrations of biocide are used, the chance of resistance developing increases. To improve the bioactivity of chemical biocides, industries have used surfactants, e.g., cetyltrimethylammonium bromide (CTAB), sodium dodecyl sulfate (SDS) in combination with chemical biocides to control and eradicate biofilm growth (Cloete et al., 1998; Simões et al., 2006).

## 3 Synthetic surfactants as anti-biofilm and anti-fouling agents

The control of biofilms using only biocides is not sufficient (Sriyutha Murthy and Venkatesan, 2008). Therefore, surfactants are combined with chemical biocides and complex-forming substances to target van der Waals and electrostatic interactions within the biofilm network (Cloete et al., 1998; Simões et al., 2006). Surfactants are regularly used to control biofilm regrowth and persistence in industrial water systems due to their solubilizing, surface wetting and penetrating properties (Simões et al., 2005). The chemical structure of surfactants can alter the surface properties of submerged structures leading to the detachment of microorganisms and the inability to form biofilms (Simões et al., 2006). Many synthetic surfactants are available as commercial formulations used to disrupt biofilms in industrial water systems (Table 1). Chemical surfactants are classified according to the nature of their hydrophilic component and are discussed below. Chemical surfactants include cationic, anionic, non-ionic and zwitterionic surfactants.

**TABLE 1:** Commercial synthetic compounds formulated to combat biofilms in industrial water systems. Adapted from Sprecher and Getsinger (2000), Azeredo et al. (2003), Simões et al. (2005), Glomski (2015).

Name	Formulations	Application	Mode of action
BULAB^®^ 6002/6086	Water-soluble liquid 60% polymeric quaternary ammonium	The products are used as microbicide which controls microorganisms in commercial and industrial recirculating cooling water towers	The products contain positively charged nitrogen atoms that bind and adsorb on microbial surfaces with a negative charge through electrostatic or charge-charge interactions
Calgon H-130M	H-130M; 50% didecyldimethylammonium chloride; liquid	It is used in once-through and industrial cooling water systems	Coating with Calgon H-130M, which contains poly-QAC (polyquat), allows biocidal activity (surfactant action). This is through adsorption on organic matter, sediments, and negatively charged surfaces
MEXEL^®^ 432/0	The liquid formulation contains 1.7% active ingredient	They are used for the protection and treatment of water-cooling circuits	Adsorbs on exposed surfaces such as wetted metal and glass to form a protective film on internal components. The aliphatic nature of the product then protects these surfaces from corrosion by limiting microbial adherence
Clam-Trol^TM^	The liquid formulation is provided in four classes of Clam-Trol CT-1,2,3,4 containing different concentrations of *n*-alkyl dimethylbenzyl ammonium chloride	They are used to control microbial growth in once-through, auxiliary/service water, wastewater, and industrial cooling water systems	The compounds have varying carbon chain lengths that work by quick absorption into anionic sediments and substrates
MACROTROL 9210	The product is presented as a liquid formulation of MACROTROL™ 9210 and NALCO^®^ 9380 containing different concentrations of dimethyl benzyl ammonium chloride	The products are useful in recirculating, auxiliary, and once-through cooling water systems	The product has corroding properties that interfere with membrane composition and respiration of microfoulants such as bacteria, fungi, and algae
VeliGON	The product is presented in liquid formulations which contain varying concentrations of dimethyl diallyl ammonium chloride	The compounds are approved for use in potable water treatment plant systems	The compound has coagulating and flocculating properties. This works by producing a dense floc that prevents the settling of zebra mussel veligers
Slimicide™ C-74	The liquid formulation contains 8% alkyl dimethylbenzyl ammonium chloride and 5% dodecylguanidine hydrochloride	The product is applicable in cooling water, recirculation, industrial cooling, and wastewater systems.	The compound destroys and loosens bacterial slimes. The product also kills slime-producing organisms such as slime molds, bacteria, fungi, and algae
H-130 Microbiocide	The product is present in liquid formulation containing didecyl dimethyl ammonium chloride	The product is applicable in once-through and recirculating cooling water systems	The product destroys the membrane composition microfoulants such as bacteria, fungi, and algae
Decont-A	This product is available in a liquid formulation containing Quaternary ammonium	This product is used to reduce the bacterial load in water cooling systems, effluent water, and sewer effluent	Decont-A disrupts biofilm as the positive charge on the ammonium ion allows it to impact and penetrate cell walls
Decont-X	This product is available in a liquid formulation containing Quaternary ammonium	Specifically formulated as surface cleaning disinfectants	Rapidly kills bacteria species as the positive charge on the ammonium ion allows it to impact and penetrate cell walls
CTAB	The product is available in solid formulation containing Quaternary ammonium	The product is applicable for treating industrial wastewater	The cationic surfactant forms micelles in liquid solution and could reduce *Pseudomonas fluorescens* biofilms by interfering with cellular respiratory activity. This results in the inactivation of the bacteria within the biofilms
SDS	The product is available as liquid or solid formulation containing sulphates	The product is used for industrial cleaning applications	The product removes all adhering bacteria by altering the cell surface properties. This occurs as SDS adsorbs to the cell surface through its polar end, exposing the non-polar end which is hydrophobic

CTAB, cetyltrimethylammonium bromide; QAC, quaternary ammonium compound; SDS, sodium dodecyl sulfate.

Sulphonates, CTAB and quaternary ammonium surfactants (QASs) are the most used cationic surfactants in industrial water systems (Cloete et al., 1998). QASs, a sub-group of quaternary ammonium compounds are particularly effective as anti-fouling agents in industrial water cooling towers (Sprecher and Getsinger, 2000; Simoes et al., 2005a). The positive charge on their alkyl chloride allows QAS’s to bind to the negatively charged microbial cells, leading to cell wall stress, lysis, and death (McDonnell and Russell, 1999).

Anionic surfactants are strong detergents but are not considered effective antimicrobial compounds. When dissolved in aqueous solvents, the hydrophilic group of anionic surfactants usually dissociates from a counter ion, and the solution becomes negatively charged due to free energy of the hydrophobic interaction. This influences detergent action as the foulants disperse and allow for easy removal in wash water (Azeredo et al., 2003). Sodium dodecyl sulfate is the most prominent anionic surfactant which weakens the biofilm cohesive forces and disrupts hydrophobic interactions within the biofilm-matrix (Simoes et al., 2005b). Meanwhile, non-ionic surfactants form part of low-temperature detergents, dispersants, and emulsifiers and their hydrophilic group does not dissociate from a counter ion when dissolved in aqueous solution. These surfactants display low toxicity to biofilm cells and include products of chemical origin such as polysorbates, polyalkylene glycols, various tween’s, ortho-phthalaldehyde, sodium hypochlorite, sodium hydroxide, and poloxamers (Simoes et al., 2005a; Percival et al., 2019). The last group, zwitterionic surfactant, has negative and positive charges on their hydrophilic end. The charges can neutralize each other depending on acidity of the solution (Hussain et al., 2020). This is the least reported group for biofilm removal, though a report showed that a combination of citric acid and zwitterionic surfactant is effective for *Staphylococcus aureus* biofilm eradication (Valentine et al., 2011).

Despite successful application of chemical surfactants in industrial treatment regimens, their usage harms the environment (Palmer and Hatley, 2018). These molecules often scatter uniformly through Brownian motion and unfavorably attach to surfaces of substrates, resulting in even higher concentrations and can therefore lead to toxicity issues (Sun et al., 2018). The sequence of surfactant toxicity initializes from its very production, discharge, and consequent introduction to the ecosystem. Chemical surfactants are often discharged to wastewater treatment facilities and are subsequently exposed to other water bodies where they can create problems if they persist for long periods, prompting the bioaccumulation of possibly unsafe or otherwise toxic substances (Rebello et al., 2013). Other manifestations of the presence of these compounds are seen in soil, plants, fauna and microbes in aquatic systems (Romanelli et al., 2004). Also, they have deleterious effects on various beneficial microbial consortia in the environment, such as DNA damage and cell lysis (Klebensberger et al., 2006; Rebello et al., 2013).

To overcome some of these disadvantages, bio-based products that have improved biodegradability and are more environmentally compatible need to be developed. Here, we’ll review current research on replacing chemically derived surfactants with biosurfactants as well as the synergy between them, a trend being implemented by many industries (Sałek and Euston, 2019), including for waste and pollution bioremediation (Ng et al., 2022).

## 4 Biosurfactants

Biosurfactants are surfactants derived from natural resources (bacteria, plants, or animals) that, like synthetic surfactants, comprise a hydrophobic and hydrophilic end (polar or non-polar) and display surface and interfacial properties (Gayathiri et al., 2022). Biosurfactants offer useful properties over their synthetic counterparts in many industrial and environmental applications (Paraszkiewicz et al., 2021). These include de/emulsification, gelling, spreading, foaming, lower critical micellar concentration, detergency, wetting and penetrating activities, ability to withstand extreme conditions, and bioactive properties (Paraszkiewicz et al., 2021). Biosurfactants are classified according to the chemical structure of the hydrophilic head group into four main types: (1) glycolipids, (2) fatty acids, (3) lipopeptides, and (4) polymers (Gayathiri et al., 2022). Generally, biosurfactants are low molecular weight compounds that can reduce surface and interfacial tension or high molecular weight compounds/polymers that serve as emulsifiers. The differentiation of biosurfactants according to their molecular weight and structural groups, as well as characterization techniques and discussions of the producer strains are reviewed and summarized elsewhere (Pacwa-Plociniczak et al., 2011; Disha and Sahasrabudhe, 2018; Baccile and Poirier, 2022; Zompra et al., 2022).

**FIGURE 2:**
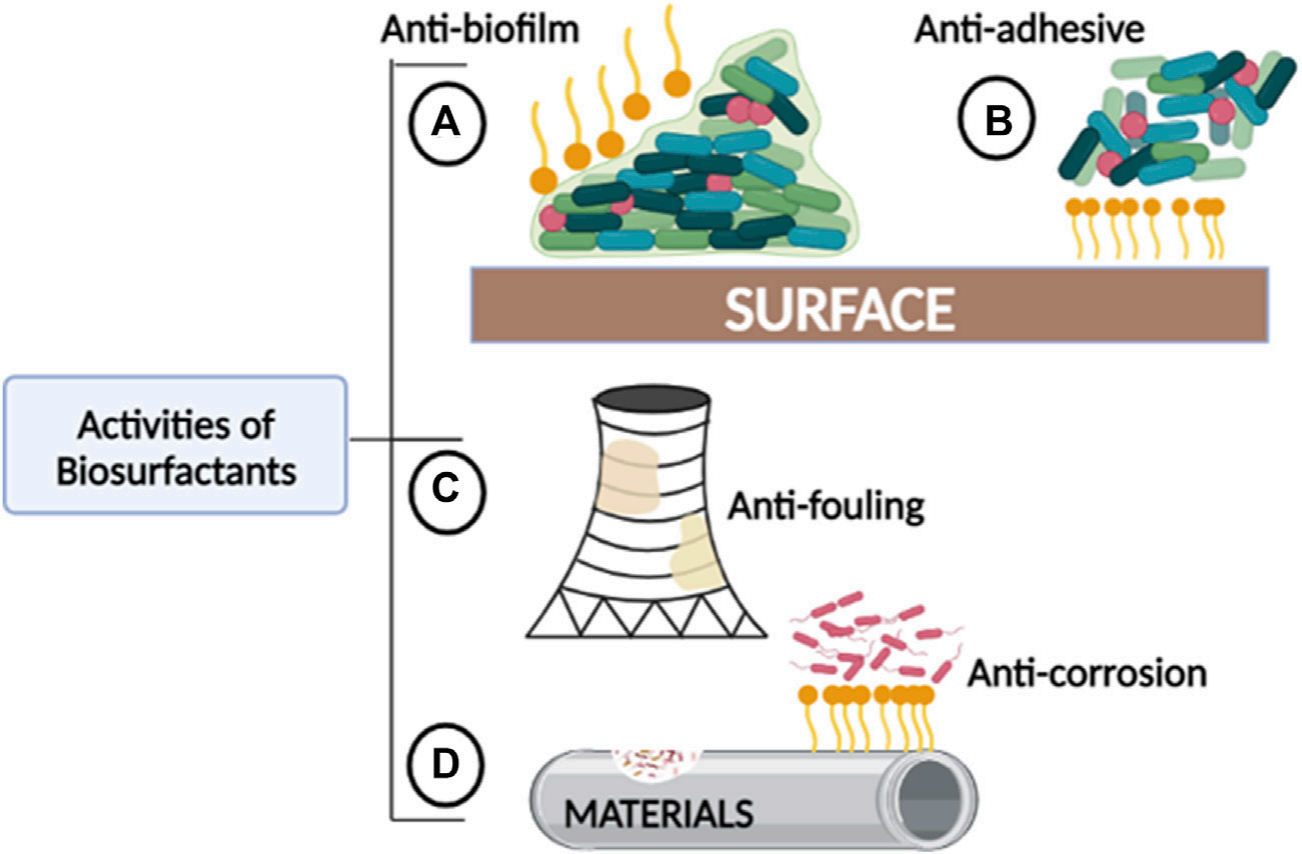
The roles played by biosurfactants in areas where fouling and corrosion commonly occur. **(A)** Anti-biofilm: Biosurfactants can prevent biofilm formation and further disrupt established biofilms. **(B)** Anti-adhesive: Biosurfactants can reduce contact or binding between bacteria and other microorganisms to abiotic surfaces. **(C)** Anti-fouling: Biosurfactants can prevent the accumulation of unwanted materials created by biofilm-fouled solid surfaces. **(D)** Anti-corrosion: Biosurfactants can prevent accelerated deterioration of metallic structures and other materials.

The structure and chemical properties of biosurfactants significantly affect their association and interactions with biofilms, and as a result, they interfere with biofilm formation through different mechanisms. Properties such as surface tension, micelle formation, cell surface hydrophobicity, emulsification, and dispersion make them suitable agents in industrial water systems. These properties enable biosurfactants to act as anti-biofilm, -adhesive, -fouling, and -corrosion agents (Figure 2). Biosurfactants penetrate and adsorb to the interface in liquid-liquid or liquid-solid interactions, decreasing the cohesion, which either prevents the attachment of biofilm-forming organisms or promotes their detachment entirely. Due to micelle formation and emulsifying properties, biosurfactants can disturb the biofilm by forming a protective layer or stable emulsion on hydrophobic surfaces. In some cases, biosurfactants can alter surface tension and hydrophobicity which causes interference in desorption processes and microbial adhesion (Das et al., 2009).

### 4.1 Biosurfactants as anti-biofilm agents

**FIGURE 3:**
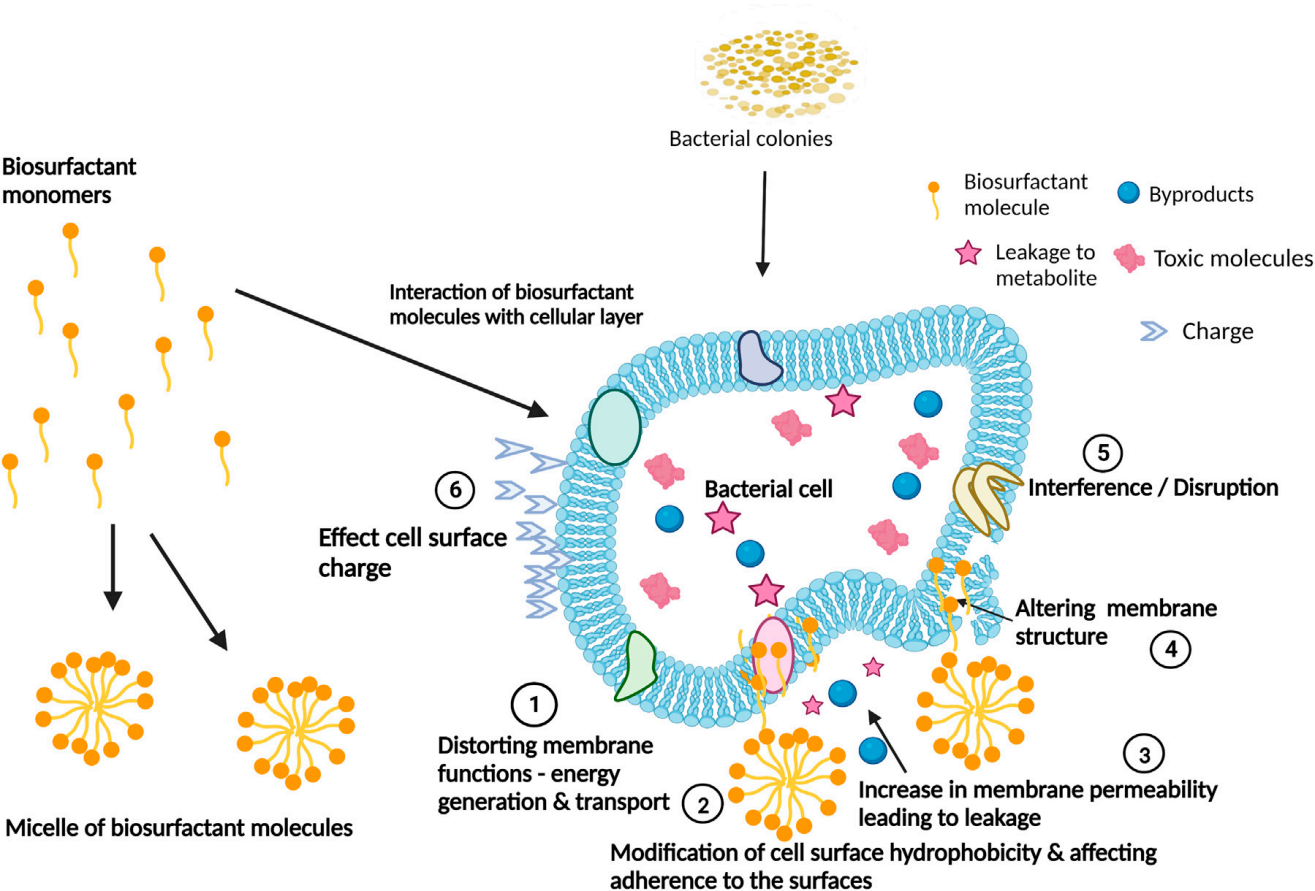
Biosurfactants’ mechanisms of action for the interference and disruption of biofilm formation. Adapted from Satpute et al. (2016). Created with BioRender.com.

Biosurfactants can be effectively introduced to industrial water systems by replicating methods for the removal of biofilms using conventional chemical surfactants (Table 2); several of these have been assessed in this regard (Table 3). Introducing biosurfactant molecules in solid or liquid form to biofilm-polluted sites could lead to biofilm dissociation by disrupting the water, nutrient and gaseous exchange channels. Biosurfactants can have intrinsic antimicrobial properties and therefore act as biocides in their own right (Płaza and Achal, 2020) or as dispersants of naturally derived compounds (Figure 3). In other words, biosurfactants can either kill the monolayers of cells after disruption or disrupt the mature biofilm by dispersion leaving the core cells alive. Lipopeptide 6-2 is a clear example of a biosurfactant that can act as killing agent as well as a dispersant agent (Song et al., 2016).

**TABLE 2:** Biosurfactants and corresponding properties that make them good anti-biofilm agents in industrial water systems.

Biosurfactant class	Functional characteristics	Surface-active properties that could make them suitable for industrial water systems	Main findings when tested/assessed as anti-biofilm agents	References
Lipopeptide (surfactin)	The structural composition has more than three surfactin molecules joined with C13, C14 and C15 fatty acid chain lengths	At 500 μg/mL critical micelle concentration, a surface tension of 26 mN/m was reached	The biosurfactant in combination with at least one biocide (pre-conditioning), prevents biofilm formation on abiotic and biotic surfaces	Ceri et al. (2013)
Lipopeptide (Fengycin A and B)	The structural composition includes molecules of C14 to C18 fatty acids chain lengths	The biosurfactant has stability at wide pH range and salt concentration (up to 10%; halotolerant)	The biosurfactant in combination with at least one biocide (pre-conditioning), prevents biofilm formation on abiotic and biotic surfaces	Ceri et al. (2013)
Glycolipid (Rhamnolipid)	Two structural compositions were characterized as a mixture of two congeners termed R1 (C_26_H_48_0_9_) and R2 (C3_2_H_58_0_13_)	The biosurfactants formed emulsion	The biosurfactants were formulated and coated with paints as a dispersant, emulsifier, or biocidal replacement	Sadasivan (2015)
Glycolipid (Sophorolipid)	The sophorolipid was characterized as lactone or diacetyl lactone form consisting of fatty acid (saturated or unsaturated) and a sugar head			
Glycolipid	The study revealed a glycolipid-like biosurfactant with about 89.5% C-18 octadecanoic acid characterized as the major fatty acid	Good emulsification and surface activity	A formulation matrix containing a mixture of biosurfactants was applied on metal panels. This allows for the protection of immersed surfaces	Soares da Silva et al. (2017), Silva et al. (2019)
		Critical micelle concentration was achieved at 600 mg/L		
		The biosurfactant was stable against high pH, temperature, and salinity		
Lipopeptide (Surfactin)	The biosurfactant was characterized as C14 to C17 surfactin homologues	Good surface tension reduction.	The biosurfactant was effective as a disinfectant as it disrupted biofilms on different surfaces	Singh and Sharma (2020)
		Critical micelle concentration was achieved at 40 mg/L		
		The biosurfactant was stable against extreme pH, temperature, and hard water conditions		
Not mentioned	Not mentioned	Not mentioned	The methods described are (1), application of biosurfactant alone or in solution on equipment surface, (2) While in operation, biosurfactant is concentrated into liquid steam within the electroporating system, and (3) circulation of biosurfactant through an electrocoat process before a biocide is added. This enhances the breakdown and cleaning of biofilms	Contos et al. (2008)
Not mentioned	Not mentioned	The biosurfactant possesses good surface and emulsifying activity	The biosurfactant was used as an additive for paint formulations for submergible surfaces	Tapia et al. (2017)
Not mentioned	An anionic biosurfactant was classified	The lowest surface tension and critical micelle concentration achieved were 27.4 mN/m, and 1.1% (v/v), respectively	The electrode surfaces of stainless steel were immersed in biosurfactant solution. Due to absorption, the biosurfactant was able to delay the corrosion of metallic surfaces	Meylheuc et al. (2001), Dagbert et al. (2006)
Not mentioned	An anionic biosurfactant was classified	The lowest surface tension and critical micelle concentration achieved were 27.4 mN/m, and 1.1% (v/v) respectively	Surface conditioning of AISI 304 stainless steel and polytetrafluoroethylene was achieved through immersion in biosurfactant solutions. The prior adsorption contributes to a significant reduction in microbial adhesion	Meylheuc et al. (2001)

**TABLE 3:** Biosurfactants as anti-biofilm agents.

Producer strain	Biosurfactant class	Dosage	Biofilm-producing strains	Main findings	Reference
*Bacillus licheniformis* VS.16, *Bacillus subtilis* VSG4	Lipopeptide	5 mg/mL	*Bacillus cereus* ATCC 11778, *Salmonella typhimurium* ATCC 19430, *Staphylococcus aureus* ATCC 29523	There was 63.9%–80.03% dispersal effect for VSG4 biosurfactant and 61.1%–68.4% for VS.16 biosurfactant against the tested strains	Giri et al. (2019)
*Pandorea pnomenusa* MS5	Exopolysaccharides	0.25 mg/mL	*Burkholderia cepacia*	*Burkholderia cepacia* biofilm was inhibited	Sacco et al. (2019)
*Bacillus subtilis* #309	Surfactin-C15	960 μg/mL	*Candida albicans*	Inhibition of about 85% of the biofilms formed	Janek et al. (2020)
*Nocardia* sp.	Biosurfactant	50–200 μg/mL	*Pseudomonas aeruginosa* ATCC 27853	There was 85% biofilm removal, indicating a dose-dependent relationship	Javadi et al. (2021)
*Candida sphaerica* UCP 0995	Lunasan	0.625–10 mg/mL	*Pseudomonas aeruginosa, Streptococcus agalactiae, Streptococcus sanguis* 12	There was 80%–92% anti-adhesive activity against the tested strains	Luna et al. (2011)
*Burkholderia thailandensis* E264	Rhamnolipids	0.39–12.5 mg/mL	*Neisseria mucosa, Actinomyces naeslundii, Streptococcus sanguinis, Streptococcus oralis,*	50%–90% inhibition of biofilms formed	Elshikh et al. (2017)
*Halomonas* sp. (BOB-3)	Rhamnolipid	125 μg/mL	*Vibrio cholerae, Salmonella typhi*)	There was 99.8% and 99.5% anti-biofilm activity on *Salmonella typhi* and *Vibrio cholerae,* respectively	Kayanadath et al. (2019)
*Candida bombicola* ATCC22214	Sophorolipids	5% (v/v)	*S. aureus* ATCC 9144, *Bacillus subtilis* BBK006	Disrupt biofilms at concentrations more than 5% (v/v) by inducing the death of planktonic cells	Diaz De Rienzo et al. (2015)
*Bacillus circulans*	Lipopeptide	10 g/L	*Serratia marcescens, Salmonella typhimurium, Proteus vulgaris, Citrobacter freundii, Micrococcus flavus Klebsiella aerogenes, Escherichia coli, Alcaligenes faecalis*	Biofilm dislodging of 59%–94% was achieved for the tested trains	Das et al. (2009)
*Bacillus subtilis*	Surfactin	0.1% and 0.5% (w/v) concentration	*Listeria monocytogenes, Staphylococcus aureus, Salmonella enteritidi*	The finding showed 95.9% disruption of preformed biofilms after 2 h contact at 0.1% surfactin	Zezzi do Valle Gomes and Nitschke (2012)
*Pseudomonas aeruginosa*	Rhamnolipids	0.25% and 1.0% (w/v) concentration		Rhamnolipid at 0.25% concentration was able to disrupt 58.5% of preformed biofilms after 2 h contact	
*Serratia marcescens* GQ214001	Glycolipid	0.0125–25 mg/mL	*Candida albicans, Pseudomonas aeruginosa and Bacillus pumilus*	The glycolipid biosurfactant mediated the disruption of the preformed biofilms of the microbial strains tested. The compound also showed 95%–99% anti-adhesive activity	Dusane et al. (2011)
*Lactobacillus pentosus, Lactobacillus paracasei*	Glycolipopeptide	0.02–25 mg/mL	*Streptococcus pyogenes, Escherichia coli, Streptococcus agalactiae, Candida albicans, Staphylococcus aureus, Pseudomonas aeruginosa*	Anti-adhesion against all the microbial strains was observed except for *E. coli* and *C. albicans*	Vecino et al. (2018)
*Acinetobacter indicus* M6	Glycolipoprotein	500 μg/mL	MRSA	There was 82.5% biofilm removal from the surface	Peele Karlapudi et al. (2018)
*Bacillus safensis* F4	Surfactin	6.25 mg/mL	*Staphylococcus epidermidis*	There was 80% anti-adhesive activity against the tested strain	Abdelli et al. (2019)
*Candida lipolytica* UCP 0988	Rufisan	0.75–12 mg/L	*Streptococcus mutans* HG*, Streptococcus agalactiae, Streptococcus mutans, Streptococcus sanguis *12, *Streptococcus oralis *J22, *Streptococcus agalactiae, Streptococcus mutans *NS	Rufisan biosurfactant displayed anti-adhesive activity on most microbial strains tested	Rufino et al. (2011)
*Lactobacillus agilis* CCUG 31450	Glycoprotein	960 mg/L	*Staphylococcus aureus*	There was anti-adhesive activity against *Staphylococcus aureus*	Gudiña et al. (2015)
*Pseudomonas fluorescens* BD5	Pseudofactin II	0.035–0.5 mg/mL	*Proteus mirabilis*, *Escherichia coli, Staphylococcus epidermidis*, *Enterococcus hirae, Candida albicans*, *Enterococcus faecalis*, *Proteus mirabilis*	Pre-treatment of the surface inhibited microbial adhesion by 36%–90%. Furthermore, a biofilm dispersal rate of 26%–70% was achieved on the tested strains	Janek et al. (2012)
*Datura stramonium*	Glycopeptide	64–1.0 μg/mL	*Candida albicans*	Eradicate biofilms formed by *C. albicans*	Mandal (2012)
*Candida sphaerica* UCP 0995	Lunasan	0.625–10 mg/mL	*Pseudomonas aeruginosa, Streptococcus agalactiae, Streptococcus sanguis* 12	There was 80%–92% anti-adhesive activity on the tested microbial strains	Luna et al. (2011)
*Lactobacillus* sp. CV8LAC	Biosurfactant	2,500–78 μg/mL	*Candida albicans* (CA-2894 and DSMZ 11225)	The biosurfactant compound has anti-biofilm potential against the tested biofilms-producing strains	Fracchia et al. (2010)
Coral *Acropora digitifera*	Biosurfactant	100 μg/mL	*P. aeruginosa* ATCC10145	Anti-biofilm activity of the biosurfactant against the tested strain	Padmavathi and Pandian (2014)
*Lactobacillus rhamnosus*	Biosurfactant components	500–0.24 mg/mL	*Acinetobacter baumannii*	There was significant inhibitory effect on cells and biofilm of *A. baumannii*	Al-Shamiri et al. (2023)
*Bacillus niabensis*	Biosurfactant	30, 50, and 100 μg/mL	*Pseudomonas stutzeri*	*B. niabesis* reduced biofilm formation by disrupting the biofilm exopolysaccharide matrix	Sanchez-Lozano et al. (2023)
*Cyperus papyrus* endophyte	Biosurfactant	0.78–1.56 mg/mL	*A. baumannii*	The biosurfactant showed significant anti-biofilm activity against *A. baumannii*	Amer et al. (2023)
*Rhodococcus* sp. SP1d	Trehalolipid	25–200 mg/mL	*Pseudomonas protegens* MP12	There was no increase in biofilm growth at 200 mg/mL biosurfactant concentration	Andreolli et al. (2023)

Biosurfactants used as biocides generally target the bacterial outer and cytoplasmic membranes and are usually more active against planktonic cells. Due to their small size and chemical properties, biosurfactants can penetrate the biofilm, directly killing microorganisms (Fleming and Rumbaugh, 2017). Many chemical biocides exhibit potent bactericidal activity but are ineffective at killing bacteria within biofilms (Singh and Sharma, 2020). The ability of some biosurfactants to show both dispersing and bactericidal activity makes them potent biocides. Although literature on biosurfactants used as biocides in industrial water systems is scarce, examples of biosurfactants with both antibacterial and anti-biofilm activity exist and include biosurfactants isolated from *Lactobacillus* spp. (Sambanthamoorthy et al., 2014), rhamnolipids (Elshikh et al., 2017) and sophorolipids (Diaz De Rienzo et al., 2015). While all the biosurfactants mentioned above were tested under laboratory conditions, they all show promise for industrial use. For example, the biosurfactants produced by *L. jensenii* and *L. rhamnosus* were not only able to prevent planktonic and sessile growth but were also able to disrupt preformed biofilms indicating that these biosurfactants can be used in industrial settings (Sambanthamoorthy et al., 2014). In another example, the rhamnolipid produced by *Burkholderia thailandensis* E264 prevents biofilm formation between 50% and 90% when plastic surfaces were precoated with a mixture of long-chain rhamnolipids (Elshikh et al., 2017). The long-chain biosurfactants also disrupted preformed immature biofilms by between 50% and 80%. Additionally, the Pf495-biosurfactant produced by *Pseudomonas fluorescens* inhibits pathogenic *Listeria monocytogenes* LO28 from attaching to polytetrafluoroethylene and stainless-steel surfaces (Meylheuc et al., 2001). This is significant as both polytetrafluoroethylene and stainless steel constitute major components in treatment plants, water-cooling circuits/systems, and industrial pipes. As shown by Song and co-authors (2016), *B. amyloliquefaciens* lipopeptide 6-2 could actively disrupt and kill the monolayers of two biofilm-forming species, *Pseudomonas aeruginosa* PAO1 and *Bacillus cereus* 1A06374 (Song et al., 2016).

Biosurfactants can also be used in combination with antibiotics to improve effectiveness or through synergistic interactions. For example, preformed biofilms of *Escherichia coli* CFT073 could not be removed by *Bacillus licheniformis* V9T14 lipopeptides, but when used in combination with different antibiotics, a significant difference was observed with more than 90% (1 log_10_) biofilm reduction (Rivardo et al., 2011). In a follow-up patent report, the same *Bacillus licheniformis* V9T14 lipopeptides were used with polycide (a biocide), which further prevented the development of biofilms on abiotic and biotic surfaces (Ceri et al., 2013). Though both antibiotics and biocides may kill the free-living planktonic cells, they are not fully effective in killing organisms within a biofilm (Ceri et al., 2013). As mentioned, biosurfactants assist by penetrating the EPS and killing the monolayer cells. Although the above authors did not discuss usage in industrial water systems, we propose a similar synergistic approach as an effective manner to treat and prevent biofilm formation.

In some cases, biosurfactants bind to the cell surface or its components and alter cell-surface hydrophobicity. This prevents microbial strains from attaching to hydrophobic surfaces (Neu, 1996; Kuiper et al., 2004). Biosurfactants such as surfactin (Mireles et al., 2001), rhamnolipid (Bharali et al., 2013) and a cyclic lipopeptide isolated from *Bacillus amyloliquefaciens* AR2 (Satpute et al., 2016) change the cell surface hydrophobicity of bacteria affecting the integrity of the cellular membrane and interfering with cell adhesion (Shakerifard et al., 2009). Rhamnolipids also act on the biofilm matrix by forming micelles within the biofilm (Paraszkiewicz et al., 2021). The micelles affect the cell surface charge and hydrophobicity within the biofilm, thereby disrupting biofilm adhesion and permeabilizing the biofilm surface (Sotirova et al., 2008; Sotirova et al., 2009). Treatment of biofilms with surfactin leads to disruption of the crystalline/semi-crystalline packing of lipid bilayers in the bacterial cell membrane (Brasseur et al., 2007). This results in dissipation of the proton motive force while interrupting the electron transport chain (Sheppard et al., 1991; Rautela et al., 2014).

Biofilm dispersal is achieved when surfactants alter the cell surface tension properties of microorganisms, thus preventing adhesion to other microbial cells as well as surfaces. Many commercialized biodispersants highlighted in literature are often surfactant-based (Guilhen et al., 2017). They function by breaking and suspending biofilms in bulk water, and the resulting clumps are then removed from the system (Cooper, 2021). When compared to their synthetic counterparts, research on biosurfactant molecules that disperse biofilms in industrial water systems is not extensive (Fleming and Rumbaugh, 2017); however, their potential as dispersing agents by mixing with other compounds or solvents was highlighted by Cao (2015). Biosurfactants as dispersing agents can prevent biofilm formation by prior coating of equipment and attachment surfaces, thus altering the surface hydrophobicity or by dispersing the biofilm through the formation of channels within the biofilm matrix (Friedlander et al., 2019).

Some biosurfactants, such as rhamnolipids cause dispersal by disrupting the biofilm matrix and solubilizing components of the matrix (Pamp and Tolken-Nielsen, 2007). The surfactin from *Bacillus tequilensis* SDS21 showed promising activity in hard water and was able to remove biofilms grown on glass, stainless steel and polystyrene, indicating that this biosurfactant might be able to disperse biofilms that form in water pipes (Singh and Sharma, 2020). Additionally, the surfactin was still viable when exposed to boiling temperatures (100°C) for 3 h and an extreme pH range (pH 5–12) (Singh and Sharma, 2020), indicating that it will still be viable when used in harsher conditions such as those found in industrial wastewater treatment centres. Another biosurfactant produced by the *Bacillus* strain AR2 was also able to maintain its anti-biofilm activity under extreme conditions. The biomolecule inhibited biofilm formation at a 46%–100% efficiency rate while also dispersing 25%–100% of *Candida* matured biofilms (Rautela et al., 2014). Di Pippo et al. (2017) analyzed the biodispersant properties of polyglucoside (a biobased surfactant) to detach mature biofilms (grown over 28 days) using lab-scale systems and artificial industrial cooling tower water. The study provided the first insights into the ability of biodispersants to eradicate unwanted biofilms in industrial cooling tower systems. Although the biocide hypochlorite showed a higher biofilm removal rate than polyglucoside (at 0.50 and 0.25 g/L), polyglucoside still removed biofilms better than the non-green formulations such as Tween^®^ 80 and Chimec 7464. Also, they found that green bio-dispersants could perform better at higher concentrations when applied to biofilms grown in batch conditions. This is in comparison to semicontinuous experiments that favoured lower concentrations, with 58%–69% biofilm dispersal values achieved (Di Pippo et al., 2017).

Quorum sensing plays an essential role in the expression of virulent genes associated with biofilm formation and is yet another target for disruption. Biosurfactants isolated from *Cobetia* sp. MM1IDA2H-1 can interfere with quorum sensing signals related to biofilm formation in the fish pathogen *Aeromonas salmonicida* (Ibacache-Quiroga et al., 2013). The proposed mechanism here is ‘signal hijacking’ as production of the purple pigment, violacein, by the test organism used in these studies (*Chromobacterium violaceum*) is under quorum sensing control. The loss of the purple phenotype is associated with the biosurfactant out-competing the native *C. violaceum* quorum sensing signalling molecules (acyl-homoserine lactone-like compounds; 3-hydroxy fatty acids (Ibacache-Quiroga et al., 2013). The binding of the mimic, as opposed to the true signal, does not lead to induction of the quorum sensing pathway and consequently the absence of the pigment.

Anti-biofilm peptides including glycopeptides, lipopeptides, and cyclic peptides possess broad-spectrum activities that target the “biofilm lifestyle” (Pletzer et al., 2016; Pletzer and Hancock, 2016) which include bacterial membranes, adhesion organelles and molecules, biofilm structural composition, and the matrix components (Pletzer and Hancock, 2016). These peptides have successfully been used to disrupt biofilms produced by ESKAPE and non-ESKAPE pathogens on surfaces (known nosocomial pathogens exhibiting multidrug resistance and virulence) (Rajput and Kumar, 2018). Peptide-based surfactants can also induce changes in gene expression leading to inhibition of biofilm formation and ultimately cell death (de la Fuente-Nunez et al., 2012). This is demonstrated by cationic peptide 1037 against *Listeria monocytogenes, Pseudomonas aeruginosa*, and *Burkholderia cenocepacia*. The peptide targets and suppresses the multiple genes responsible for biofilm formation (de la Fuente-Nunez et al., 2012). Later, de la Fuente-Nunez et al. (2014) described a potent anti-biofilm peptide 1018 that induces a cellular response by binding, blocking, and degrading (p)ppGpp [guanosine 3′,5′-bis(pyrophosphate)], also known as the magic spot, which serves as an important signal and secondary messenger molecule in biofilm development. Interfering with the (p)ppGpp ultimately leads to the dispersal of biofilms (de la Fuente-Nunez et al., 2014). When developing biosurfactant formulations for use in industrial water systems, the focus should be complete disruption or prevention of biofilm formation to increase plant efficiency and eliminate or decrease the need for biocide use in industrial systems.

### 4.2 Application of biosurfactants in industrial water systems as anti-fouling and anti-corrosion agents

The accumulation of unwanted organic materials on any surface is called biofouling (Coetser and Cloete, 2005). Biofouling in industrial water systems involves microorganisms (e.g., bacteria, fungi, yeasts, moulds, diatoms, or algae) and macroorganisms such as mussels, protozoans, and barnacles (Melo and Bott, 1997). Biosurfactants used in anti-fouling strategies target biofilms that create dead biomass. Industrial biofouling in water systems results in several problems that include decreased membrane flux, shutdown, damage, reduced heat-exchanger efficiency, blockages, and energy loss (Coetser and Cloete, 2005). Many extreme conditions occur in industrial systems, thus the applied biosurfactants must have properties such as hard water resistance, stability, and ability to withstand harsh conditions such as high -acidic/alkaline conditions, temperature, and salinity. Other properties such as solubilizing properties, good wetting, interfacial action, and the ability to disrupt hydrophobicity between bacteria and surfaces are important for the successful removal of biofilms in industrial water systems (Sulaimon and Adeyemi, 2018). As discussed earlier, microbial fouling of industrial water systems can have severe health and economic consequences. Although most biosurfactant research focuses on treating biofilms in healthcare setups, we propose that biosurfactants can also play a big role in treating industrial wastewater since numerous biosurfactants can remove mature biofilms and prevent biofilm formation.

One approach to combat fouling problems is to mix paints with biosurfactants before applying them on surfaces. This improves resistance to fouling by modifying surface properties such as hydrophobicity, surface tension and charge. However, this strategy is challenging to implement as the dispersal and leach rate of the compound must be controlled and a coating mechanism that enables the gradual release of the compound needs development (Yebra et al., 2004). Aleman-Vega et al. (2020) reported a 70% biofouling decrease by degreasing and covering acrylic plates with paint formulated with biosurfactants isolated from *Bacillus niabensis* (My-30), *B. niabensis* (S-69) or *Ralstonia* sp. (S-74). The *Bacillus amyloliquefaciens* anti-CA lipopeptide also showed anti-fouling potential as the biosurfactant killed the *Balanus amphitrite* larvae and inhibited the growth of protozoans (Song et al., 2016), which are two organisms that contribute to fouling in cooling towers (Critchley and Bentham, 2007; Mizhir, 2012).

In addition to anti-fouling activities, biosurfactants can act as anti-corrosion agents (Table 4). For example, the biosurfactant alkylpolyglucoside inhibits corrosion of 907 steel and the efficacy could be altered by changing the alkyl chain lengths (Du et al., 2004). Another anti-biocorrosion strategy is the combination of biosurfactants and polymeric substances such as epoxy resins, polyester, polyaspartate, alginate, and polyglutamate. In a study by Zin et al. (2018), a biosurfactant complex containing rhamnolipids and an alginate-based biopolymer produced extracellularly by *Pseudomonas* sp. PS-17 inhibited the corrosion of aluminium alloy D16T successfully. A two-to four-fold increase in the repassivation kinetics was observed for the treated surfaces, and the effects were attributed to the rhamnolipids in the complex (Zin et al., 2018). Since microbially induced corrosion is a consequence of biofilm formation, efforts should be tailored towards preventing microbial contamination of metallic components of industrial water systems (Grasland et al., 2003; Silva et al., 2018). Metal is prone to biocorrosion and constitutes major components of industrial water systems such as water-cooling circuits, towers, vacuum pumps, treatment plants, pipes, and sensors. *Bacillus* species are well known for producing biosurfactants capable of combating biofilm-induced corrosion (Purwasena et al., 2019). Certain *Bacillus* spp. produce various surfactants that inhibit the growth of bacteria that induce metal surface corrosion (Jayaraman et al., 1999; Santos et al., 2016). Finally, the *Pseudomonas stutzeri* F01 biosurfactant is an eco-friendly biocide tested in the oil and gas industry, where 30%–40% of the corrosion problems are attributed to microbial corrosion (Parthipan et al., 2018). The authors report these biosurfactant compounds as efficient microbial inhibitors because they possess anti-biofilm properties against corrosive bacterial strains even at low concentrations.

**TABLE 4:** Examples of biosurfactants used against biocorrosion.

Producer strain	Class	Activity	Mode of action	References
*Pseudomonas fluorescens*	Biosurfactant	Inhibiting the corrosion of AISI 304 stainless steel.	The biosurfactant adsorption was able to facilitate the inactivation of the oxide layer and act as barrier to the diffusion of chlorides and dissolved oxygen	Dagbert et al. (2006)
*Pseudomonas* sp. PS-17	Rhamnolipid	Inhibiting the corrosion of alloy	Adsorption of the rhamnolipid biosurfactant on the alloy surface allows the modification of the oxide film layer, thereby increasing corrosion resistance	Zin et al. (2018)
*Pseudomonas mosselii* F01	Biosurfactant	Significant inhibition activity against corroding carbon steel (API 5LX) corrosive bacterial strains	The mode of action is attributed to the adsorption of the biosurfactant functional groups over the metal surface through interfacial action of the hydrophobic and hydrophilic moiety	Parthipan et al. (2018)
*Pseudoxanthomonas* sp. F3	Rhamnolipid	Eliminate biofilms associated with biocorrosion	The rhamnolipid biosurfactant enabled hydrophobic interactions within the surface by reducing the interfacial tension. This results in surface films that prevent biocorrosion issues	Astuti et al. (2018)
*Bacillus* sp	Biosurfactant	Microbial-influenced corrosion on carbon steel ST37 was inhibited	The biosurfactant was able to penetrate the matrix and disrupt the water channels. This accelerates biofilm disruption off the steel surface	Purwasena et al. (2019)
*Bacillus* sp. H2O-1	Surfactin	Control of sulfate-reducing bacteria on examined surfaces such as carbon steel, stainless steel AISI 304; 430, polystyrene, and galvanized steel	The addition of AMS lipopeptide extract (mixture of four surfactin homologues) influenced the hydrophobicity and energy level of the examined surfaces	Korenblum et al. (2012)
*Pseudomonas* sp. PS-17	Rhamnolipids biocomplex	The surface-active products were able to inhibit the corrosion of D16T aluminium alloy in distilled water	The mechanism includes adsorption of biosurfactant molecules and formation of micelles (a multilayer organic protective film), which insulate efficiently on the metal surface	Pokhmurs’kyi et al. (2014)

## 5 Conclusion

Biosurfactants are well-known antimicrobial and anti-biofilm agents with activity recorded against numerous microbial organisms, including yeast, Gram-positive and Gram-negative bacteria. While numerous biosurfactants have been investigated for anti-biofilm properties, the most studied are rhamnolipids and lipopeptides. This is due to their prevalence and well-characterized structures. Unfortunately, reports of the use of biosurfactants in industrial water systems are lacking, specifically in areas combating biofouling and biocorrosion. Industries for whom these issues are problematic are starting to implement management practices that employ more eco-friendly products, suggesting a bright future for biosurfactants. There remains a vast number of uncharacterized amphiphilic compounds of biological origin that represent a huge untapped resource, each of which brings its own nuanced mixture of properties that could be suitable or represent a substantial improvement over the well-characterized compounds for use in applications described throughout this review. Even from the limited number of reports available, biosurfactants have proven to be effective in industrial water systems and can be used simultaneously to protect surfaces as well as reduce the effect of microbially induced corrosion or fouling. By co-formulating biocides and biosurfactants one can also significantly increase the bioactivity of the biocide, ultimately decreasing the high concentrations of biocide needed. Thus, co-formulation with biosurfactants represents a more ecological, cost-effective, and renewable solution with diminished impact when water is released into the environment. In their management programs, industrial water users could even involve biosurfactants combined with other molecules, such as polymers and bio-based surfactants, to proffer novel and safe alternatives. Given the number of novel compounds yet to be described, research activities are expected to introduce new compounds and methodologies to enable this.

## References

[B1] Abdelli F. Jardak M. Elloumi J. Stien D. Cherif S. Mnif S. (2019). Antibacterial, anti-adherent and cytotoxic activities of surfactin(s) from a lipolytic strain *Bacillus safensis* F4. Biodegradation 30 (4), 287–300. 10.1007/s10532-018-09865-4 30600423

[B2] Al-Shamiri M. M. Wang J. Zhang S. Li P. Odhiambo W. O. Chen Y. (2023). Probiotic Lactobacillus species and their biosurfactants eliminate Acinetobacter baumannii biofilm in various manners. Microbiol. Spect. 11 (2), e0461422. 10.1128/spectrum.04614-22 PMC10100725 36920192

[B3] Aleman-Vega M. Sanchez-Lozano I. Hernandez-Guerrero C. J. Hellio C. Quintana E. T. (2020). Exploring anti-fouling activity of biosurfactants producing marine bacteria isolated from Gulf of California. Int. J. Mol. Sci. 21 (17), 6068. 10.3390/ijms21176068 32842499 PMC7504147

[B4] Amer M. A. Wasfi R. Hamed S. M. (2023). Biosurfactant from Nile Papyrus endophyte with potential antibiofilm activity against global clones of Acinetobacter baumannii. Front. Cell Infect. Microbiol. 13, 1210195. 10.3389/fcimb.2023.1210195 37520441 PMC10373939

[B5] Amirinejad N. Shahriary P. Hassanshahian M. (2023). Investigation of the synergistic effect of glycolipid biosurfactant produced by Shewanella algae with some antibiotics against planktonic and biofilm forms of MRSA and antibiotic resistant Acinetobacter baumannii. World J. Microbiol. Biotechnol. 39 (2), 45. 10.1007/s11274-022-03492-1 36534183

[B6] Anderl J. N. Franklin M. J. Stewart P. S. (2000). Role of antibiotic penetration limitation in *Klebsiella pneumoniae* biofilm resistance to ampicillin and ciprofloxacin. Antimicrob. Agents Chemother. 44 (7), 1818–1824. 10.1128/aac.44.7.1818-1824.2000 10858336 PMC89967

[B7] Andreolli M. Villanova V. Zanzoni S. D'onofrio M. Vallini G. Secchi N. (2023). Characterization of trehalolipid biosurfactant produced by the novel marine strain Rhodococcus sp. SP1d and its potential for environmental applications. Microb. Cell Factories 22 (1), 126. 10.1186/s12934-023-02128-9 PMC10339595 37443119

[B8] Aquatech (2019). Industrial water: our essential guide to pollution, treatment & solutions. Available at: https://www.aquatechtrade.com/news/industrial-water/industrial-water-essential-guide#:∼:text=Industrial%20water%3A%20Our%20essential%20guide%20to%20pollution%2C%20treatment%20%26%20solutions&text=Without%20water%2C%20many%20companies%20and,production%20pr (Accessed August 14, 2023).

[B9] Ashby M. Neale J. Knott S. Critchley I. (1994). Effect of antibiotics on non-growing planktonic cells and biofilms of *Escherichia coli* . J. Antimicrob. Chemother. 33 (3), 443–452. 10.1093/jac/33.3.443 8040110

[B10] Astuti D. I. Purwasena I. A. Putri F. Z. (2018). Potential of biosurfactant as an alternative biocide to control biofilm associated biocorrosion. J. Environ. Sci. Technol. 11 (2), 104–111. 10.3923/jest.2018.104.111

[B11] Azeredo J. Pacheco A. P. Lopes I. Oliveira R. Vieira M. J. (2003). Monitoring cell detachment by surfactants in a parallel plate flow chamber. Water Sci. Technol. 47 (5), 77–82. 10.2166/wst.2003.0286 12701910

[B12] Baccile N. Poirier A. (2022). Microbial biobased amphiphiles (Biosurfactants): General aspects on CMC, surface tension and phase behaviour. Biosurfactants, Elsevier. In press. hal-03714497.

[B169] Bales P. M. Renke E. M. May S. L. Shen Y. Nelson D.C. (2013) Purification and characterization of biofilm-associated EPS exopolysaccharides from ESKAPE organisms and other pathogens. PLoS ONE. 8 (6), e67950, 10.1371/journal.pone.0067950 23805330 PMC3689685

[B13] Beech I. Bergel A. Mollica A. Flemming H. Scotto V. Sand W. (2000). Microbially influenced corrosion of industrial materials. Recent Adv. Study Bio-corrosion 2, 00–02.

[B14] Bharali P. Saikia J. P. Ray A. Konwar B. K. (2013). Rhamnolipid (RL) from *Pseudomonas aeruginosa* OBP1: a novel chemotaxis and antibacterial agent. Colloids Surf. B Biointerfaces 103, 502–509. 10.1016/j.colsurfb.2012.10.064 23261573

[B15] Brasseur R. Braun N. El Kirat K. Deleu M. Mingeot-Leclercq M.-P. Dufrêne Y. F. (2007). The biologically important surfactin lipopeptide induces nanoripples in supported lipid bilayers. Langmuir 23 (19), 9769–9772. 10.1021/la7014868 17696376

[B172] Cao T. (2015). Generation of biodispersants for offshore oil spill response. Meml. Univ. Res. Repos.

[B16] Ceri H. Turner R. Martinotti M. Rivardo F. (2013). Biosurfactant composition produced by a new Bacillus licheniforms strain, uses and products thereof. Google Patents. Available at: https://patents.google.com/patent/US8377455B2/en .

[B17] Cloete E. Flemming H.-C. (2012). “Environmental impact of cooling water treatment for biofouling and biocorrosion control,” in Operational and environmental consequences of large industrial cooling water systems (Boston, MA: Springer), 303–314.

[B18] Cloete T. E. Jacobs L. Brözel V. S. (1998). The chemical control of biofouling in industrial water systems. Biodegradation 9 (1), 23–37. 10.1023/a:1008216209206 9807802

[B19] Coetser S. E. Cloete T. E. (2005). Biofouling and biocorrosion in industrial water systems. Crit. Rev. Microbiol. 31 (4), 213–232. 10.1080/10408410500304074 16417202

[B20] Colagiorgi A. Bruini I. Di Ciccio P. Zanardi E. Ghidini S. Ianieri A. (2017). *Listeria monocytogenes* biofilms in the wonderland of food Industry. Pathogens 6, 41. 10.3390/pathogens6030041 28869552 PMC5617998

[B21] Colvin K. M. Irie Y. Tart C. S. Urbano R. Whitney J. C. Ryder C. (2012). The Pel and Psl polysaccharides provide *Pseudomonas aeruginosa* structural redundancy within the biofilm matrix. Environ. Microbiol. 14 (8), 1913–1928. 10.1111/j.1462-2920.2011.02657.x 22176658 PMC3840794

[B22] Contos M. A. Bourdeau M. J. Pillar L. L. (2008). Treating an electrocoat system with a biosurfactant. Google Patents.

[B23] Cooper J. (2021). “Biocide dosing and bio dispersants in cooling tower water treatment”. Availabe at: https://www.bvwater.co.uk/b-v-water-news/biocides-bio-dispersants-cooling-tower-water-treatment . [Accessed, 14 August 2023].

[B24] Costa J. A. Treichel H. Santos L. O. Martins V. G. (2018). “Solid-state fermentation for the production of biosurfactants and their applications,” in Current developments in biotechnology and bioengineering (Elsevier), 357–372.

[B25] Critchley M. Bentham R. (2007). Legionella and protozoa in cooling towers: implications for public health and chemical control. Environ. Health 7 (2), 36–44.

[B26] Dagbert C. Meylheuc T. Bellon-Fontaine M.-N. (2006). Corrosion behaviour of AISI 304 stainless steel in presence of a biosurfactant produced by *Pseudomonas fluorescens* . Electrochim. Acta. 51 (24), 5221–5227. 10.1016/j.electacta.2006.03.063

[B27] Danese P. N. Pratt L. A. Kolter R. (2000). Exopolysaccharide production is required for development of *Escherichia coli* K-12 biofilm architecture. J. Bacteriol. 182 (12), 3593–3596. 10.1128/jb.182.12.3593-3596.2000 10852895 PMC101973

[B28] Das P. Mukherjee S. Sen R. (2009). Antiadhesive action of a marine microbial surfactant. Colloids Surf. B Biointerfaces. 71 (2), 183–186. 10.1016/j.colsurfb.2009.02.004 19285837

[B29] Das T. Sharma P. K. Busscher H. J. Van Der Mei H. C. Krom B. P. (2010). Role of extracellular DNA in initial bacterial adhesion and surface aggregation. Appl. Environ. Microbiol. 76 (10), 3405–3408. 10.1128/aem.03119-09 20363802 PMC2869138

[B30] De Giani A. Zampolli J. Di Gennaro P. (2021). Recent trends on biosurfactants with antimicrobial activity produced by bacteria associated with human health: different perspectives on their properties, challenges, and potential applications. Front. Microbiol. 12, 655150. 10.3389/fmicb.2021.655150 33967992 PMC8104271

[B31] De La Fuente-Nunez C. Korolik V. Bains M. Nguyen U. Breidenstein E. B. Horsman S. (2012). Inhibition of bacterial biofilm formation and swarming motility by a small synthetic cationic peptide. Antimicrob. Agents Chemother. 56 (5), 2696–2704. 10.1128/aac.00064-12 22354291 PMC3346644

[B32] De La Fuente-Nunez C. Reffuveille F. Haney E. F. Straus S. K. Hancock R. E. (2014). Broad-spectrum anti-biofilm peptide that targets a cellular stress response. PLoS Pathog. 10 (5), e1004152. 10.1371/journal.ppat.1004152 24852171 PMC4031209

[B33] Di Pippo F. De Tora F. Di Gregorio L. Buccolini M. Capocecera R. Rossetti S. (2017). Green bio-dispersant removal efficacy estimation for controlling biofilms in cooling towers. Ann. Microbiol. 67, 779–784. 10.1007/s13213-017-1301-3

[B34] Di Pippo F. Di Gregorio L. Congestri R. Tandoi V. Rossetti S. (2018). Biofilm growth and control in cooling water industrial systems. FEMS Microbiol. Ecol. 94 (5), fiy044. 10.1093/femsec/fiy044 29596620

[B35] Diaz De Rienzo M. A. Banat I. M. Dolman B. Winterburn J. Martin P. J. (2015). Sophorolipid biosurfactants: possible uses as antibacterial and antibiofilm agent. Nat. Biotechnol. 32 (6), 720–726. 10.1016/j.nbt.2015.02.009 25738966

[B36] Diederen B. (2008). Legionella spp. and Legionnaires’ disease. J. Infect. 56 (1), 1–12. 10.1016/j.jinf.2007.09.010 17980914

[B37] Disha S. S. Sahasrabudhe S. (2018). Biosurfactant: classification, properties and recent application in cosmetic. J. Emerg. Technol. Innov. Res. 5 (10), 160–167.

[B38] Dongari-Bagtzoglou A. (2008). Pathogenesis of mucosal biofilm infections: challenges and progress. Expert Rev. anti-infect. Ther. 6 (2), 201–208. 10.1586/14787210.6.2.201 18380602 PMC2712878

[B39] Donlan R. M. (2001). Biofilm formation: a clinically relevant microbiological process. Clin. Infect. Dis. 33 (8), 1387–1392. 10.1086/322972 11565080

[B40] Doroshenko N. Tseng B. S. Howlin R. P. Deacon J. Wharton J. A. Thurner P. J. (2014). Extracellular DNA impedes the transport of vancomycin in *Staphylococcus epidermidis* biofilms preexposed to subinhibitory concentrations of vancomycin. Antimicrob. Agents Chemother. 58 (12), 7273–7282. 10.1128/aac.03132-14 25267673 PMC4249571

[B41] Du M. Gao R. J. Gong P. Wang Q. Z. (2004). The new-generation of green surfactant (alkylpolyglucoside) as an inhibitor to the corrosion of 907 steel in seawater. Chin. Chem. Lett. 15 (8), 985–988.

[B42] Dueholm M. S. Søndergaard M. T. Nilsson M. Christiansen G. Stensballe A. Overgaard M. T. (2013). Expression of Fap amyloids in *Pseudomonas aeruginosa, P. fluorescens*, and *P. putida* results in aggregation and increased biofilm formation. Microbiologyopen 2 (3), 365–382. 10.1002/mbo3.81 23504942 PMC3684753

[B43] Dusane D. H. Pawar V. S. Nancharaiah Y. V. Venugopalan V. P. Kumar A. R. Zinjarde S. S. (2011). Anti-biofilm potential of a glycolipid surfactant produced by a tropical marine strain of *Serratia marcescens* . Biofouling 27 (6), 645–654. 10.1080/08927014.2011.594883 21707248

[B44] Elshikh M. Funston S. Chebbi A. Ahmed S. Marchant R. Banat I. M. (2017). Rhamnolipids from non-pathogenic *Burkholderia thailandensis* E264: physicochemical characterization, antimicrobial and antibiofilm efficacy against oral hygiene related pathogens. N. Biotechnol. 36, 26–36. 10.1016/j.nbt.2016.12.009 28065676

[B45] Fields B. S. Benson R. F. Besser R. E. (2002). Legionella and legionnaires’ disease: 25 years of investigation. Clin. Microbiol. Rev. 15 (3), 506–526. 10.1128/cmr.15.3.506-526.2002 12097254 PMC118082

[B46] Fleming D. Rumbaugh K. P. (2017). Approaches to dispersing medical biofilms. Microorganisms 5 (2), 15. 10.3390/microorganisms5020015 28368320 PMC5488086

[B47] Flemming H.-C. Neu T. R. Wozniak D. J. (2007). The EPS matrix: the “house of biofilm cells”. J. Bacteriol. 189 (22), 7945–7947. 10.1128/jb.00858-07 17675377 PMC2168682

[B48] Fracchia L. Cavallo M. Allegrone G. Martinotti M. (2010). A Lactobacillus-derived biosurfactant inhibits biofilm formation of human pathogenic *Candida albicans* biofilm producers. Appl. Microbiol. Biotechnol. 2, 827–837.

[B49] Friedlander A. Nir S. Reches M. Shemesh M. (2019). Preventing biofilm formation by dairy-associated bacteria using peptide-coated surfaces. Front. Microbiol. 10, 1405. 10.3389/fmicb.2019.01405 31297098 PMC6608603

[B50] Gayathiri E. Prakash P. Karmegam N. Varjani S. Awasthi M. K. Ravindran B. (2022). Biosurfactants: potential and eco-friendly material for sustainable agriculture and environmental safety—a review. Agronomy 12 (3), 662. 10.3390/agronomy12030662

[B51] Giri S. S. Ryu E. C. Sukumaran V. Park S. C. (2019). Antioxidant, antibacterial, and anti-adhesive activities of biosurfactants isolated from *Bacillus* strains. Microb. Pathog. 132, 66–72. 10.1016/j.micpath.2019.04.035 31028863

[B52] Gloag E. S. Turnbull L. Huang A. Vallotton P. Wang H. Nolan L. M. (2013). Self-organization of bacterial biofilms is facilitated by extracellular DNA. Proc. Natl. Acad. Sci. 110 (28), 11541–11546. 10.1073/pnas.1218898110 23798445 PMC3710876

[B53] Glomski L. (2015). Zebra mussel chemical control guide. US Army Corps of Engineers: Waterways Experiment Station.

[B54] Grasland B. Mitalane J. Briandet R. Quemener E. Meylheuc T. Linossier I. (2003). Bacterial biofilm in seawater: cell surface properties of early-attached marine bacteria. Biofouling 19 (5), 307–313. 10.1080/0892701031000121041 14650085

[B55] Gudiña E. J. Fernandes E. C. Teixeira J. A. Rodrigues L. R. (2015). Antimicrobial and anti-adhesive activities of cell-bound biosurfactant from *Lactobacillus agilis* CCUG31450. RSC Adv. 5 (110), 90960–90968. 10.1039/c5ra11659g

[B56] Guilhen C. Forestier C. Balestrino D. (2017). Biofilm dispersal: multiple elaborate strategies for dissemination of bacteria with unique properties. Mol. Microbiol. 105 (2), 188–210. 10.1111/mmi.13698 28422332

[B57] Hussain S. M. S. Mahboob A. Kamal M. S. (2020). Synthesis and evaluation of zwitterionic surfactants bearing benzene ring in the hydrophobic tail. Mater. (Basel) 13 (8), 1858. 10.3390/ma13081858 PMC7215865 32326587

[B58] Ibacache‐Quiroga C. Ojeda J. Espinoza‐Vergara G. Olivero P. Cuellar M. Dinamarca M. (2013). The hydrocarbon-degrading marine bacterium*Cobetia*sp. strain MM1IDA2H-1 produces a biosurfactant that interferes with quorum sensing of fish pathogens by signal hijacking: biosurfactant quorum sensing signal hijacking. Microb. Biotechnol. 6 (4), 394–405. 10.1111/1751-7915.12016 23279885 PMC3917474

[B59] Janek T. Drzymala K. Dobrowolski A. (2020). *In vitro* efficacy of the lipopeptide biosurfactant surfactin-C15 and its complexes with divalent counterions to inhibit *Candida albicans* biofilm and hyphal formation. Biofouling 36 (2), 210–221. 10.1080/08927014.2020.1752370 32292058

[B60] Janek T. Łukaszewicz M. Krasowska A. (2012). Antiadhesive activity of the biosurfactant pseudofactin II secreted by the Arctic bacterium *Pseudomonas fluorescens* BD5. BMC Microbiol. 12 (1), 24–29. 10.1186/1471-2180-12-24 22360895 PMC3310744

[B61] Javadi A. Pourmand M. R. Hamedi J. Gharebaghi F. Baseri Z. Mohammadzadeh R. (2021). Evaluation of anti-biofilm potential of biosurfactant extracted from *Nocardia* species. Folia Med. Plovdiv. 63 (3), 392–399. 10.3897/folmed.63.e54386 34196143

[B62] Jayaraman A. Hallock P. Carson R. Lee C.-C. Mansfeld F. Wood T. (1999). Inhibiting sulfate-reducing bacteria in biofilms on steel with antimicrobial peptides generated *in situ* . Appl. Microbiol. Biotechnol. 52 (2), 267–275. 10.1007/s002530051520 10499267

[B64] Johnson L. Horsman S. R. Charron-Mazenod L. Turnbull A. L. Mulcahy H. Surette M. G. (2013). Extracellular DNA-induced antimicrobial peptide resistance in *Salmonella enterica serovar Typhimurium* . BMC Microbiol. 13, 115–118. 10.1186/1471-2180-13-115 23705831 PMC3724500

[B65] Justo J. Bookstaver P. (2014). Antibiotic lock therapy: review of technique and logistical challenges. Infect. Drug Resist 7, 343–363. 10.2147/idr.s51388 25548523 PMC4271721

[B66] Kaplan J. B. (2010). Biofilm dispersal: mechanisms, clinical implications, and potential therapeutic uses. J. Dent. Res. 89 (3), 205–218. 10.1177/0022034509359403 20139339 PMC3318030

[B67] Kaplan J. B. Velliyagounder K. Ragunath C. Rohde H. Mack D. Knobloch J. K.-M. (2004). Genes involved in the synthesis and degradation of matrix polysaccharide in *Actinobacillus actinomycetemcomitans* and *Actinobacillus pleuropneumoniae* biofilms. J. Bacteriol. 186 (24), 8213–8220. 10.1128/jb.186.24.8213-8220.2004 15576769 PMC532409

[B68] Kayanadath S. Nathan V. K. Ammini P. (2019). Anti-biofilm activity of biosurfactant derived from *Halomonas* sp., a Lipolytic marine bacterium from the Bay of Bengal. Microbiology 88 (5), 585–599. 10.1134/s0026261719050072

[B69] Keren I. Shah D. Spoering A. Kaldalu N. Lewis K. (2004). Specialized persister cells and the mechanism of multidrug tolerance in *Escherichia coli* . J. Bacteriol. 186 (24), 8172–8180. 10.1128/jb.186.24.8172-8180.2004 15576765 PMC532439

[B70] Klebensberger J. Rui O. Fritz E. Schink B. Philipp B. (2006). Cell aggregation of *Pseudomonas aeruginosa* strain PAO1 as an energy-dependent stress response during growth with sodium dodecyl sulfate. Archives Microbiol. 185 (6), 417–427. 10.1007/s00203-006-0111-y 16775748

[B71] Korber D. R. Lawrence J. R. Sutton B. Caldwell D. E. (1989). Effect of laminar flow velocity on the kinetics of surface recolonization by Mot+ and Mot− *Pseudomonas fluorescens* . Microb. Ecol. 18, 1–19. 10.1007/bf02011692 24196017

[B72] Korenblum E. De Araujo L. V. Guimarães C. R. De Souza L. M. Sassaki G. Abreu F. (2012). Purification and characterization of a surfactin-like molecule produced by *Bacillus* sp. H2O-1 and its antagonistic effect against sulfate reducing bacteria. BMC Microbiol. 12 (252). 10.1186/1471-2180-12-252 PMC3577442 23131170

[B167] Kosaric N. (2001) Biosurfactants and their application for soil bioremediation. Food Technol. Biotechnol. 39 (4) 295-304. ISSN 1330-9862.

[B73] Kuiper I. Lagendijk E. L. Pickford R. Derrick J. P. Lamers G. E. Thomas‐Oates J. E. (2004). Characterization of two *Pseudomonas putida* lipopeptide biosurfactants, putisolvin I and II, which inhibit biofilm formation and break down existing biofilms. Mol. Microbiol. 51 (1), 97–113. 10.1046/j.1365-2958.2003.03751.x 14651614

[B74] Kusnetsov J. M. Martikainen P. J. Jousimies-Somer H. R. Väisänen M.-L. Tulkki A. I. Ahonen H. E. (1993). Physical, chemical and microbiological water characteristics associated with the occurrence of *Legionella* in cooling tower systems. Water Res. 27 (1), 85–90. 10.1016/0043-1354(93)90198-q

[B75] Lewenza S. (2013). Extracellular DNA-induced antimicrobial peptide resistance mechanisms in *Pseudomonas aeruginosa* . Front. Microbiol. 4, 21. 10.3389/fmicb.2013.00021 23419933 PMC3572637

[B76] Lewis K. (2007). Persister cells, dormancy and infectious disease. Nat. Rev. Microbiol. 5 (1), 48–56. 10.1038/nrmicro1557 17143318

[B77] Liu Y. Zhang W. Sileika T. Warta R. Cianciotto N. P. Packman A. I. (2011). Disinfection of bacterial biofilms in pilot-scale cooling tower systems. Biofouling 27 (4), 393–402. 10.1080/08927014.2011.577525 21547755 PMC4507511

[B78] Luna J. M. Rufino R. D. Sarubbo L. A. Rodrigues L. R. Teixeira J. A. De Campos-Takaki G. M. (2011). Evaluation antimicrobial and antiadhesive properties of the biosurfactant Lunasan produced by *Candida sphaerica* UCP 0995. Curr. Microbiol. 62 (5), 1527–1534. 10.1007/s00284-011-9889-1 21327556

[B79] Lynch D. J. Fountain T. L. Mazurkiewicz J. E. Banas J. A. (2007). Glucan-binding proteins are essential for shaping *Streptococcus mutans* biofilm architecture. FEMS Microbiol. Lett. 268 (2), 158–165. 10.1111/j.1574-6968.2006.00576.x 17214736 PMC1804096

[B80] Maaike C. De Vires E. Vaughan E. Kleerebezem M. De Vos W. (2006). *Lactobacillus plantarum* survival, functional and potential probiotic properties in the human intestinal tract. Int. Dairy J. 16 (9), 1018–1028. 10.1016/j.idairyj.2005.09.003

[B81] Makhlouf A. S. H. Botello M. A. (2018). “Failure of the metallic structures due to microbiologically induced corrosion and the techniques for protection,” in Handbook of materials failure analysis (Elsevier), 1–18.

[B82] Mandal S. M. (2012). A novel hydroxyproline rich glycopeptide from pericarp of datura stramonium: proficiently eradicate the biofilm of antifungals resistant *Candida albicans* . Biopolymers 98 (4), 332–337. 10.1002/bip.22083 23193597

[B83] Mcdonnell G. Russell A. D. (1999). Antiseptics and disinfectants: activity, action, and resistance. Clin. Microbiol. Rev. 12 (1), 147–179. 10.1128/cmr.12.1.147 9880479 PMC88911

[B84] Melo L. Bott T. (1997). Biofouling in water systems. Exp. Therm. fluid Sci. 14 (4), 375–381. 10.1016/s0894-1777(96)00139-2

[B85] Meylheuc T. Van Oss C. J. Bellon-Fontaine M. N. (2001). Adsorption of biosurfactant on solid surfaces and consequences regarding the bioadhesion of *Listeria monocytogenes* LO28. J. Appl. Microbiol. 91 (5), 822–832. 10.1046/j.1365-2672.2001.01455.x 11722659

[B86] Mireles J. R. Toguchi A. Harshey R. M. (2001). *Salmonella enterica serovar typhimurium* swarming mutants with altered biofilm-forming abilities: surfactin inhibits biofilm formation. J. Bacteriol. 183 (20), 5848–5854. 10.1128/jb.183.20.5848-5854.2001 11566982 PMC99661

[B87] Mizhir A. H. (2012). Influence of copper on larval and adult phases densities of the barnacle Balanus amphitrete amphitrite on the cooling system water supply of Al-Najibiyah thermal power plant/Iraq. J. Purity, Util. React. Environ. 2, 85–91.

[B88] Muhammad M. H. Idris A. L. Fan X. Guo Y. Yu Y. Jin X. (2020). Beyond risk: bacterial biofilms and their regulating approaches. Front. Microbiol. 11, 928. 10.3389/fmicb.2020.00928 32508772 PMC7253578

[B89] Mulcahy H. Charron M. Lewenza S. (2008). Extracellular DNA chelates cations and induces antimicrobial resistance in *Escherichia coli* biofilms. PLoS Pathog. 4, 1213–1218. 10.1371/journal.ppat.1000213 PMC2581603 19023416

[B90] Neu T. R. (1996). Significance of bacterial surface-active compounds in interaction of bacteria with interfaces. Microbiol. Rev. 60 (1), 151–166. 10.1128/mr.60.1.151-166.1996 8852899 PMC239423

[B91] Ng Y. J. Chan S. S. Khoo K. S. Munawaroh H. S. H. Lim H. R. Chew K. W. (2023). Recent advances and discoveries of microbial-based glycolipids: prospective alternative for remediation activities. Biotechnol. Adv. 68, 108198. 10.1016/j.biotechadv.2023.108198 37330152

[B92] Ng Y. J. Lim H. R. Khoo K. S. Chew K. W. Chan D. J. C. Bilal M. (2022). Recent advances of biosurfactant for waste and pollution bioremediation: substitutions of petroleum-based surfactants. Environ. Res. 212, 113126. 10.1016/j.envres.2022.113126 35341755

[B166] Okuda K. Nagahori R. Yamada S. Sugimoto S. Sato C. Sato M. (2018) The composition and structure of biofilms developed by Propionibacterium acnes Isolated from cardiac pacemaker devices. Front. Microbiol. 9 (182), 10.3389/fmicb.2018.00182 PMC5817082 29491850

[B93] O’toole G. Kaplan H. B. Kolter R. (2000). Biofilm formation as microbial development. Annu. Rev. Microbiol. 54, 49–79. 10.1146/annurev.micro.54.1.49 11018124

[B94] Pacwa-Plociniczak M. Plaza G. A. Piotrowska-Seget Z. Cameotra S. S. (2011). Environmental applications of biosurfactants: recent advances. Int. J. Mol. Sci. 12 (1), 633–654. 10.3390/ijms12010633 21340005 PMC3039971

[B95] Padmavathi A. R. Pandian S. K. (2014). Antibiofilm activity of biosurfactant producing coral associated bacteria isolated from gulf of mannar. Indian J. Microbiol. 54 (4), 376–382. 10.1007/s12088-014-0474-8 25320434 PMC4186942

[B96] Palmer J. Flint S. Brooks J. (2007). Bacterial cell attachment, the beginning of a biofilm. J. Ind. Microbiol. Biotechnol. 34 (9), 577–588. 10.1007/s10295-007-0234-4 17619090

[B97] Palmer M. Hatley H. (2018). The role of surfactants in wastewater treatment: impact, removal and future techniques: A critical review. Water Res. 147, 60–72. 10.1016/j.watres.2018.09.039 30300782

[B173] Pamp S. J. Tolker-Nielsen T. (2007) Multiple roles of biosurfactants in structural biofilm development by Pseudomonas aeruginosa. J. Bacteriol. 189 (6), 2531 – 2539. 10.1128/JB.01515-06 17220224 PMC1899385

[B98] Paraszkiewicz K. Moryl M. Plaza G. Bhagat D. Satpute K. S. Bernat P. (2021). Surfactants of microbial origin as antibiofilm agents. Int. J. Environ. Health Res. 31 (4), 401–420. 10.1080/09603123.2019.1664729 31509014

[B99] Parthipan P. Sabarinathan D. Angaiah S. Rajasekar A. (2018). Glycolipid biosurfactant as an eco-friendly microbial inhibitor for the corrosion of carbon steel in vulnerable corrosive bacterial strains. J. Mol. Liq. 261, 473–479. 10.1016/j.molliq.2018.04.045

[B100] Peele Karlapudi A. Venkateswarulu T. C. Srirama K. Kota R. K. Mikkili I. Kodali V. P. (2018). Evaluation of anti-cancer, antimicrobial and anti-biofilm potential of biosurfactant extracted from an *Acinetobacter* M6 strain. J. King Saud. Univ. Sci. 32 (1), 223–227. 10.1016/j.jksus.2018.04.007

[B101] Percival S. L. Mayer D. Kirsner R. S. Schultz G. Weir D. Roy S. (2019). Surfactants: role in biofilm management and cellular behaviour. Int. Wound J. 16 (3), 753–760. 10.1111/iwj.13093 30883044 PMC7948769

[B102] Percival S. L. Suleman L. Vuotto C. Donelli G. (2015). Healthcare-associated infections, medical devices and biofilms: risk, tolerance and control. J. Med. Microbiol. 64 (4), 323–334. 10.1099/jmm.0.000032 25670813

[B103] Pereira R. P. A. Peplies J. Hofle M. G. Brettar I. (2017). Bacterial community dynamics in a cooling tower with emphasis on pathogenic bacteria and Legionella species using universal and genus-specific deep sequencing. Water Res. 122, 363–376. 10.1016/j.watres.2017.06.011 28622629

[B104] Płaza G. Achal V. (2020). Biosurfactants: eco-friendly and innovative biocides against biocorrosion. Int. J. Mol. Sci. 21 (6), 2152. 10.3390/ijms21062152 32245097 PMC7139319

[B105] Pletzer D. Coleman S. R. Hancock R. E. (2016). Anti-biofilm peptides as a new weapon in antimicrobial warfare. Curr. Opin. Microbiol. 33, 35–40. 10.1016/j.mib.2016.05.016 27318321 PMC5069134

[B106] Pletzer D. Hancock R. E. (2016). Antibiofilm peptides: potential as broad-spectrum agents. J. Bacteriol. 198 (19), 2572–2578. 10.1128/jb.00017-16 27068589 PMC5019066

[B107] Pokhmurs’kyi V. І. Karpenko О. V. Zin І. М. Tymus М. B. Veselivs’ka H. H. (2014). Inhibiting action of biogenic surfactants in corrosive media. Mat. Sci. 50 (3), 448–453. 10.1007/s11003-014-9741-4

[B108] Prigent-Combaret C. Vidal O. Dorel C. Lejeune P. (1999). Abiotic surface sensing and biofilm-dependent regulation of gene expression in *Escherichia coli* . J. Bacteriol. 181 (19), 5993–6002. 10.1128/jb.181.19.5993-6002.1999 10498711 PMC103626

[B109] Purwasena I. A. Astuti D. I. Fauziyyah N. A. Putri D. a. S. Sugai Y. (2019). Inhibition of microbial influenced corrosion on carbon steel ST37 using biosurfactant produced by *Bacillus* sp. Mat. Res. Express 6 (11), 115405. 10.1088/2053-1591/ab4948

[B110] Rabin N. Zheng Y. Opoku-Temeng C. Du Y. Bonsu E. Sintim H. O. (2015). Biofilm formation mechanisms and targets for developing antibiofilm agents. Future Med. Chem. 7 (4), 493–512. 10.4155/fmc.15.6 25875875

[B111] Rajput A. Kumar M. (2018). “Anti-biofilm peptides: a new class of quorum quenchers and their prospective therapeutic applications,” in Biotechnological applications of quorum sensing inhibitors (Springer), 87–110.

[B112] Rao T. S. (2012). “Microbial fouling and corrosion: fundamentals and mechanisms,” in Operational and environmental consequences of large industrial cooling water systems (Boston, MA: Springer), 95–126.

[B114] Rautela R. Singh A. K. Shukla A. Cameotra S. S. (2014). Lipopeptides from *Bacillus* strain AR2 inhibits biofilm formation by *Candida albicans* . Ant. Van Leeuwenhoek 105 (5), 809–821. 10.1007/s10482-014-0135-2 24623107

[B115] Rebello S. Asok A. K. Mundayoor S. Jisha M. S. (2013). “Surfactants: chemistry, toxicity and remediation,” in Pollutant diseases, remediation and recycling (Springer Science & Business Media), 277–320.

[B116] Reid A. N. Whitfield C. (2005). Functional analysis of conserved gene products involved in assembly of *Escherichia coli* capsules and exopolysaccharides: evidence for molecular recognition between wza and wzc for colanic acid biosynthesis. J. Bacteriol. 187 (15), 5470–5481. 10.1128/jb.187.15.5470-5481.2005 16030241 PMC1196018

[B117] Renner L. D. Weibel D. B. (2011). Physicochemical regulation of biofilm formation. MRS Bull. 36 (5), 347–355. 10.1557/mrs.2011.65 22125358 PMC3224470

[B118] Rivardo F. Martinotti M. G. Turner R. J. Ceri H. (2011). Synergistic effect of lipopeptide biosurfactant with antibiotics against *Escherichia coli* CFT073 biofilm. Int. J. Antimicrob. Agents 37 (4), 324–331. 10.1016/j.ijantimicag.2010.12.011 21316197

[B171] Romanelli M. F. Moraes M. C. F. Villavicencio A. L. C. H. Borrely S. I. (2004) Evaluation of toxicity reduction of sodium dodecyl sulfate submitted to electron beam radiation. Radiation physics and Chemistry. 71 (1), 409 – 411. 10.1016/j.radphyschem.2004.03.038

[B119] Romero D. Aguilar C. Losick R. Kolter R. (2010). Amyloid fibers provide structural integrity to *Bacillus subtilis* biofilms. Proc. Natl. Acad. Sci. 107 (5), 2230–2234. 10.1073/pnas.0910560107 20080671 PMC2836674

[B120] Rosenberg M. Bayer E. A. Delarea J. Rosenberg E. (1982). Role of thin fimbriae in adherence and growth of *Acinetobacter calcoaceticus* RAG-1 on hexadecane. Appl. Environ. Microbiol. 44 (4), 929–937. 10.1128/aem.44.4.929-937.1982 16346118 PMC242119

[B121] Rufino R. D. Luna J. M. Sarubbo L. A. Rodrigues L. R. Teixeira J. A. Campos-Takaki G. M. (2011). Antimicrobial and anti-adhesive potential of a biosurfactant Rufisan produced by *Candida lipolytica* UCP 0988. Colloids Surf. B Biointerfaces 84 (1), 1–5. 10.1016/j.colsurfb.2010.10.045 21247740

[B122] Sacco L. P. Castellane T. C. L. Polachini T. C. De Macedo Lemos E. G. Alves L. M. C. (2019). Exopolysaccharides produced by *Pandoraea* shows emulsifying and anti-biofilm activities. J. Polym. Res. 26 (4), 91. 10.1007/s10965-019-1737-1

[B123] Sadasivan L. (2015). Aqueous coatings and paints incorporating one or more antimicrobial biosurfactants and methods for using same. Google Patents.

[B124] Sałek K. Euston S. R. (2019). Sustainable microbial biosurfactants and bioemulsifiers for commercial exploitation. Process Biochem. 85, 143–155. 10.1016/j.procbio.2019.06.027

[B125] Sambanthamoorthy K. Feng X. Patel R. Patel S. Paranavitana C. (2014). Antimicrobial and antibiofilm potential of biosurfactants isolated from lactobacilli against multi-drug-resistant pathogens. BMC Microbiol. 14, 197–199. 10.1186/1471-2180-14-197 25124936 PMC4236506

[B126] Sanchez-Lozano I. Munoz-Cruz L. C. Hellio C. Band-Schmidt C. J. Cruz-Narvaez Y. Becerra-Martinez E. (2023). Metabolomic insights of biosurfactant activity from Bacillus niabensis against planktonic cells and biofilm of Pseudomonas stutzeri involved in marine biofouling. Int. J. Mol. Sci. 24 (4), 4249. 10.3390/ijms24044249 36835662 PMC9965525

[B127] Santos V. Drummond R. N. Dias-Souza M. (2016). “Biosurfactants as antimicrobial and antibiofilm agents,” in Current developments in biotechnology and bioengineering: Human and animal health applications (Elsevier), 371–402.

[B128] Satpute S. K. Banpurkar G A. Banat M. I. Sangshetti N J. Patil R. Gade W. (2016). Multiple roles of biosurfactants in biofilms. Curr. Pharm. Des. 22 (11), 1429–1448. 10.2174/1381612822666160120152704 26786675

[B129] Savage V. J. Chopra I. O’neill A. J. (2013). *Staphylococcus aureus* biofilms promote horizontal transfer of antibiotic resistance. Antimicrob. Agents. Chemother. 57 (4), 1968–1970. 10.1128/aac.02008-12 23357771 PMC3623343

[B130] Shakerifard P. Gancel F. Jacques P. Faille C. (2009). Effect of different *Bacillus subtilis* lipopeptides on surface hydrophobicity and adhesion of *Bacillus cereus* 98/4 spores to stainless steel and Teflon. Biofouling 25 (6), 533–541. 10.1080/08927010902977943 19431000

[B131] Sharma D. Misba L. Khan A. U. (2019). Antibiotics versus biofilm: an emerging battleground in microbial communities. Antimicrob. Resist. Infect. Control 8 (1), 76–10. 10.1186/s13756-019-0533-3 31131107 PMC6524306

[B132] Sheppard J. Jumarie C. Cooper D. Laprade R. (1991). Ionic channels induced by surfactin in planar lipid bilayer membranes. Biochim. Biophys. Acta - Biomembr. 1064 (1), 13–23. 10.1016/0005-2736(91)90406-x 1709052

[B133] Shineh G. Mobaraki M. Perves Bappy M. J. Mills D. K. (2023). Biofilm formation, and related impacts on healthcare, food processing and packaging, industrial manufacturing, marine industries, and sanitation–A review. Appl. Microbiol. 3 (3), 629–665. 10.3390/applmicrobiol3030044

[B134] Silva M. Almeida D. Da Silva R. S. Meira H. Almeida F. Fernandes M. (2018). Chemically modified natural substances screening for biofilms inhibition and biofouling control. Chem. Eng. Trans. 64, 655–660. 10.3303/CET1864110

[B135] Silva M. Medeiros A. Almeida D. Meira H. Almeida F. Silva R. (2019). Anti-fouling protection of surfaces immersed in marine environment by natural surfactants as bioactive contained in coating based on natural Resin. Chem. Eng. Trans. 74, 1507–1512. 10.3303/CET1974252

[B136] Simoes M. Pereira M. O. Vieira M. J. (2005a). Action of a cationic surfactant on the activity and removal of bacterial biofilms formed under different flow regimes. Water Res. 39 (2-3), 478–486. 10.1016/j.watres.2004.09.018 15644256

[B137] Simões M. Pereira M. O. Vieira M. J. (2005). “Control of biofilms using surfactants: persistence and regrowth,” in Biofilms: Persistence and ubiquity (Manchester: Biofilm Club), 369–338.

[B138] Simoes M. Pereira M. O. Vieira M. J. (2005b). Effect of mechanical stress on biofilms challenged by different chemicals. Water Res. 39 (20), 5142–5152. 10.1016/j.watres.2005.09.028 16289205

[B139] Simões M. Simões L. C. Machado I. Pereira M. O. Vieira M. J. (2006). Control of flow-generated biofilms with surfactants. Food Bioprod. process. 84 (4), 338–345. 10.1205/fbp06022

[B140] Singh A. K. Sharma P. (2020). Disinfectant-like activity of lipopeptide biosurfactant produced by *Bacillus tequilensis* strain SDS21. Colloids Surf. B Biointerfaces 185, 110514. 10.1016/j.colsurfb.2019.110514 31639569

[B141] Soares Da Silva R. D. C. F. Almeida D. G. Meira H. M. Silva E. J. Farias C. B. B. Rufino R. D. (2017). Production and characterization of a new biosurfactant from *Pseudomonas cepacia* grown in low-cost fermentative medium and its application in the oil industry. Biocatal. Agric. Biotechnol. 12, 206–215. 10.1016/j.bcab.2017.09.004

[B168] Solano C. Echeverz M. Lasa I. (2014) Biofilm dispersion and quorum sensing. Curr. Opin. Microbiol. 18, 96–104. 10.1016/j.mib.2014.02.008 24657330

[B142] Song B. Wang Y. Z. Wang G. Y. Liu G. L. Li W. Z. Yan F. (2016). The lipopeptide 6-2 produced by *Bacillus amyloliquefaciens* anti-CA has potent activity against the biofilm-forming organisms. Mar. Pollut. Bull. 108 (1-2), 62–69. 10.1016/j.marpolbul.2016.04.062 27184127

[B143] Sotirova A. Spasova D. Vasileva-Tonkova E. Galabova D. (2009). Effects of rhamnolipid-biosurfactant on cell surface of *Pseudomonas aeruginosa* . Microbiol. Res. 164 (3), 297–303. 10.1016/j.micres.2007.01.005 17416508

[B144] Sotirova A. V. Spasova D. I. Galabova D. N. Karpenko E. Shulga A. (2008). Rhamnolipid-biosurfactant permeabilizing effects on gram-positive and gram-negative bacterial strains. Curr. Microbiol. 56 (6), 639–644. 10.1007/s00284-008-9139-3 18330632

[B145] Sprecher S. L. Getsinger K. D. (2000). Zebra mussel chemical control guide. U.S. Army Engineer Research and Development Center.

[B146] Sriyutha Murthy P. Venkatesan R. (2008). “Industrial biofilms and their control,” in Marine and industrial biofouling (Springer Berlin Heidelberg), 65–101.

[B147] Stevenson G. Andrianopoulos K. Hobbs M. Reeves P. R. (1996). Organization of the *Escherichia coli* K-12 gene cluster responsible for production of the extracellular polysaccharide colanic acid. J. Bacteriol. 178 (16), 4885–4893. 10.1128/jb.178.16.4885-4893.1996 8759852 PMC178271

[B148] Sulaimon A. A. Adeyemi B. J. (2018). “Effects of interfacial tension alteration on the destabilization of water-oil emulsions,” in Science and Technology behind nanoemulsions (Books on Demand).

[B174] Sun W. Wang Y. Zhang W. Ying H. Wang P (2018) Novel surfactant peptide for removal of biofilms. Colloids Surf. B. 172, 180-186. 10.1016/j.colsurfb.2018.08.29 30149322

[B149] Tapia M. a. D. Herrera J. R. O. Quiroga C. J. I. (2017). Strain of *Cobetia marina* and biosurfactant extract obtained from same. Google Patents.

[B150] Valentine R. Jervis-Bardy J. Psaltis A. Tan L. W. Wormald P. J. (2011). Efficacy of using a hydrodebrider and of citric acid/zwitterionic surfactant on a *Staphylococcus aureus* bacterial biofilm in the sheep model of rhinosinusitis. Am. J. Rhinol. Allergy 25 (5), 323–326. 10.2500/ajra.2011.25.3659 22186246

[B151] Vecino X. Rodriguez-Lopez L. Ferreira D. Cruz J. M. Moldes A. B. Rodrigues L. R. (2018). Bioactivity of glycolipopeptide cell-bound biosurfactants against skin pathogens. Int. J. Biol. Macromol. 109, 971–979. 10.1016/j.ijbiomac.2017.11.088 29162463

[B152] Verberk J. Vreeburg J. Rietveld L. Van Dijk J. (2009). Particulate fingerprinting of water quality in the distribution system. Water sa. 35 (2). 10.4314/wsa.v35i2.76754

[B153] Verderosa A. D. Totsika M. Fairfull-Smith K. E. (2019). Bacterial biofilm eradication agents: a current review. Front. Chem. 7, 824. 10.3389/fchem.2019.00824 31850313 PMC6893625

[B154] Walser S. M. Gerstner D. G. Brenner B. Höller C. Liebl B. Herr C. E. (2014). Assessing the environmental health relevance of cooling towers–a systematic review of legionellosis outbreaks. Int. J. Hyg. Environ. Health. 217 (2-3), 145–154. 10.1016/j.ijheh.2013.08.002 24100053

[B155] Wang H. Li Y. Yang H. Lin K. A. Shao T. Hope J. (2023). Biofilms controlling in industrial cooling water systems: A mini-review of strategies and best practices. ACS Appl. Bio Mater 6, 3213–3220. 10.1021/acsabm.3c00319 37428894

[B156] Wéry N. Bru-Adan V. Minervini C. Delgénes J.-P. Garrelly L. Godon J.-J. (2008). Dynamics of *Legionella* spp. and bacterial populations during the proliferation of *L. pneumophila* in a cooling tower facility. Appl. Environ. Microbiol. 74 (10), 3030–3037. 10.1128/aem.02760-07 18390683 PMC2394956

[B157] Whitchurch C. B. Tolker-Nielsen T. Ragas P. C. Mattick J. S. (2002). Extracellular DNA required for bacterial biofilm formation. Science 295 (5559), 1487. 10.1126/science.295.5559.1487 11859186

[B170] Williams T. McGinley H. R. (2010) Deactivation of industrial water treatment biocides. NACE – International corrosion conference series. 3, 10.20535/2218-9300932012138742

[B159] Yebra D. M. Kiil S. Dam-Johansen K. (2004). Anti-fouling technology—Past, present and future steps towards efficient and environmentally friendly anti-fouling coatings. Prog. Org. Coat. 50 (2), 75–104. 10.1016/j.porgcoat.2003.06.001

[B160] Zezzi Do Valle Gomes M. Nitschke M. (2012). Evaluation of rhamnolipid and surfactin to reduce the adhesion and remove biofilms of individual and mixed cultures of food pathogenic bacteria. Food control. 25 (2), 441–447. 10.1016/j.foodcont.2011.11.025

[B161] Zhang H. Wang J. Chang Z. Liu X. Chen W. Yu Y. (2021). *Listeria monocytogenes* contamination characteristics in two ready-to-eat meat plants from 2019 to 2020 in Shanghai. Front. Microbiol. 12, 729114. 10.3389/fmicb.2021.729114 34512606 PMC8427505

[B162] Zhang L. Mah T.-F. (2008). Involvement of a novel efflux system in biofilm-specific resistance to antibiotics. J. Bacteriol. 190 (13), 4447–4452. 10.1128/jb.01655-07 18469108 PMC2446775

[B163] Zhang X. Bishop P. L. (2003). Biodegradability of biofilm extracellular polymeric substances. Chemosphere 50 (1), 63–69. 10.1016/s0045-6535(02)00319-3 12656230

[B164] Zin I. M. Pokhmurskii V. I. Korniy S. A. Karpenko O. V. Lyon S. B. Khlopyk O. P. (2018). Corrosion inhibition of aluminium alloy by rhamnolipid biosurfactant derived from *Pseudomonas* sp. PS-17. anti-corros. Methods Mater 65 (6), 517–527. 10.1108/acmm-03-2017-1775

[B165] Zompra A. A. Chasapi S. A. Twigg M. S. Salek K. Anestopoulos I. Galanis A. (2022). Multi-method biophysical analysis in discovery, identification, and in-depth characterization of surface‐active compounds. Front. Mar. Sci. 9. 10.3389/fmars.2022.1023287

